# Gut microbiome and lung cancer: mechanisms, interactions, and dietary interventions

**DOI:** 10.1080/29933935.2025.2501313

**Published:** 2025-05-23

**Authors:** Jayashri Chakraborty, Ngashepam Lanchenba Singh, Bhrigu Kumar Das

**Affiliations:** Department of Pharmacology, School of Pharmaceutical Sciences, Girijananda Chowdhury University, Azara, Guwahati, India

**Keywords:** Gut microbiome, lung cancer, Gut-lung axis, polyphenols, probiotics

## Abstract

Lung cancer continues to claim countless lives globally. Several studies have shown that the gut microbiome is vital in maintaining healthy lung function through the gut-lung axis. A comparison between the gut microflora of healthy volunteers and lung cancer patients revealed that changes in the composition of gut microflora occur in lung cancer patients. The gut microflora may contribute to lung cancer by influencing immune reactions, inflammatory pathways, bacterial metabolites modulating host metabolism, microbiome dysbiosis, genotoxicity, virulence, and bacteria-induced epigenetic alterations. Thus, it may be assumed that maintaining a healthy gut microflora could help prevent lung cancer. Nutraceuticals are specialized products designed to support health and address specific nutritional needs. They contain ingredients like vitamins, minerals, probiotics, polyphenols, and herbs to reduce the risk or impact of certain illnesses. Nutraceuticals, including probiotics and polyphenols, play a role in preventing and treating various cancers, including lung cancer, by modulating the gut microbiome. This review examines the link between the gut microbiome and lung cancer, how it contributes to cancer development, and the impact of dietary interventions – particularly probiotics, polyphenols, and dietary fibers – on lung cancer prevention and treatment.

## Introduction

Lung cancer continues to have the highest mortality rate worldwide, causing more deaths each year than any other cancer.^1^ Though it is not a gender-specific cancer, males are more likely than females to develop and die from lung cancer over their lives.^2^ According to the American Cancer Society (ACS), lung cancer ranks as the second most prevalent cancer worldwide.^1^ The prevalence of lung cancer varies widely from country to country, influenced by factors such as smoking rates, air pollution levels, and dietary habits. The GLOBOCAN 2022 report highlights 9.7 million cancer-related deaths and 20 million new cases globally, reflecting a slight increase in cases but a marginal decline in fatalities since 2020. Lung and breast cancers remain the most diagnosed, contributing 12.4% and 11.6% of new cases, followed by colorectal (9.6%), prostate (7.3%), and stomach cancers (4.9%). Lung cancer accounts for the highest mortality rate at 18.7%, with colorectal (9.3%), liver (7.8%), breast (6.9%), and stomach cancers (6.8%) following. In 2021, approximately 2 million people were diagnosed with lung cancer worldwide, and about 1.76 million lives were lost to the disease.^3–6^ In India, lung cancer makes up 5.9% of all cancer cases and is responsible for 8.1% of all cancer-related deaths.^7^

Lung cancer is generally divided into two main categories depending on how the cancer cells appear under a microscope. Small cell lung cancer (SCLC) makes up about 18% of cases, tends to grow quickly, and often spreads to other organs. In contrast, non-small cell lung cancer (NSCLC), which accounts for around 78% of cases, usually grows and spreads at a slower pace. NSCLC itself includes three subtypes: squamous cell carcinoma (25% of cases), adenocarcinoma (about 40%), and large cell lung cancer (about 10%). Additionally, while the rates of squamous cell carcinoma and small cell carcinoma are decreasing, the occurrence of adenocarcinoma is rising in men and women alike.^8^ Although advancements in tumor biology and genomic profiling have improved the treatment of this neoplasm, these methods’ effectiveness and side effects still present significant challenges. Therefore, new interventions are necessary.^9,10^ Recent studies have uncovered a fascinating link between the digestive system and the lungs, often called the gut-lung axis, which plays a role in lung cancer development. Lungs were once considered sterile organs, but advancements in research have revealed that they host a complex microbiome that includes *Firmicutes*, *Bacteroidetes*, *Staphylococcus*, *Streptococcus*, *Lactobacillus*, along with *Proteobacteria* and *Actinobacteria*, a microbiota composition comparable to that of healthy individuals’ gut microbiota.^1,11,12^ Lung microbiota is smaller; however, not less significant than gut microbiota.^13^ The gut microbiota significantly functions in human health care administration, affecting the gastrointestinal (GI) tract and other organs.^14^ Numerous studies have found that shifts in the gut microbiome’s composition, as well as function, known as gut dysbiosis,^15^ play a vital role in disease mechanisms of GI diseases like GI tumors,^16^ inflammatory bowel disease,^17^ and also non-GI diseases like obesity,^18^ lung cancer,^19^ cardiovascular diseases,^20^ and diabetes.^21^ The disruption of the regular cell cycle of the host may result in the modification of the cell and its expression of the protein, which regulates the processes of cell division, DNA repair, programmed cell death (apoptosis), and controlling the host’s inflammatory and immune responses. These disruptions are a few actions that gut bacteria show on the host’s health and homeostasis.^22,23^ Beyond maintaining host health, gut bacteria play a pivotal role in cancer development and treatment.^24^ They significantly influence the progression and therapeutic outcomes of lung cancer, including responses to immune checkpoint therapies and chemotherapy.^25,26^ The possible mechanisms by which gut microbiota causes lung cancer include metabolism-related genotoxicity, chronic systemic inflammation and immune response.^27,28^ In lung cancer, particularly in non-small-cell lung carcinoma (NSCLC), a more diverse gut microbiome has been linked to improved immune checkpoint blockade (ICB) therapy responses. Notably, the bacterium *Bifidobacterium longum* was found to be more prevalent in responders to the therapy.^29^ This suggests that a higher diversity of gut bacteria, including specific species, can enhance anti-tumor immune responses. This relationship underscores the antitumorigenic role of the microbiota.^30,31^ According to the study by William Coley on “Coley toxins”, different bacteria or bacterial products can boost anti-cancer immunity.^32^ *Lactobacillus rhamnosus* activates neutrophils and dendritic cells and modulates the activation of T cells, leading to more decisive tumor-inhibiting actions.^33^ Microorganisms and their byproducts can enhance cytokines with tumor-fighting and immune-activating effects, like TNF and IL-12, by activating innate immunity via pathogen-associated molecular patterns. IL-22 supports tissue repair and defends against infections. Additionally, certain gut bacteria, like *Bacteroides dorei*, *Parabacteroides distasonis*, and *Paraprevotellaxylaniphila*, can enhance the generation of IFN-γ by CD8 T cells, resulting in the rise of tumor-fighting immunity in mice.^34,35^

Natural compounds may be perceived as a substitute therapy for pulmonary disorders.^36^ Numerous studies have demonstrated that various natural foods are vital in treating and managing lung cancer. These include delicious options like berries, pomegranates, apples, tomatoes, rice bran, and bitter melons. Spices like turmeric, saffron, garlic, chili, rosemary, and ginger contribute to this effort. Nutritious foods also include soy, corn, cruciferous vegetables, and mushrooms. Certain fungi like *Thelephoraganbajun* and *Calvatia gigantea* have been highlighted for their potential benefits. Incorporating these natural dietary products can be essential to lung cancer care.^37^

Nutraceuticals are bioactive phytochemicals derived from food or natural sources recognized for their potential to enhance cancer therapy. These compounds can effectively induce apoptosis in cancer cells, making them valuable adjuncts to chemotherapy. Key nutraceuticals with potent anticancer properties include stilbenes, flavonoids, carotenoids, and sulfur-containing compounds.^38^ Probiotics and polyphenols are various components of nutraceuticals that have potential effects in the treatment of lung cancer. Probiotics may improve tumor response to immune checkpoint inhibitors (ICI) by influencing the gut flora.^39^ According to multiple research studies, probiotics such as *Bifidobacterium* and *Lactobacillus reuteri* (Lr) have been shown to improve ICI therapy.^40,41^ Research from laboratory studies, animal experiments, and population studies highlights the chemo-preventive properties of dietary polyphenols in reducing the risk of cancer, including lung carcinogenesis.^42^ Despite these promising insights, the exact ways in which the gut microbiome influences lung cancer – particularly concerning disease onset, progression, and response to treatment – are still poorly understood. While there is emerging evidence of interactions between gut bacteria and the lung tumor environment, a comprehensive understanding of these complex relationships remains elusive. Additionally, dietary changes, which can alter the gut microbiome, might offer new ways to influence lung cancer outcomes. However, the effects of such dietary interventions on lung cancer have not been fully explored. Hence, keeping this view in mind, we aim to collect known research findings to explain how gut microbiota composition and function influence lung cancer carcinogenesis and therapy. We will look at how gut bacteria influences both healthy and abnormal immune responses through the gut-lung axis and how this interaction presents novel opportunities for safe and effective prevention and treatment of lung cancer. Furthermore, dietary interventions, specifically prebiotics, probiotics, and polyphenols, are highlighted as supplemental therapy for lung cancer.

## Methodology

A comprehensive search was conducted across several well-known scientific databases, including PubMed®, ResearchGate®, Google Scholar®, Scopus®(Elsevier), Cochrane Library, Web of Science® and *clinicaltrial.gov*, as well as relevant books, up to November 2024. The search was performed in English using key terms and combinations related to gut microbiota and lung cancer. Primary search terms included lung cancer, gut-lung axis, gut microbiome, human microbiome, dysbiosis, treatment, probiotics, prebiotics, nutraceuticals, animal study, *in-vitro*, *in-vivo*, antibiotics, and clinical trials or studies. Relevant studies were thoroughly reviewed, and the most critical findings were synthesized. The insights gathered from these sources were carefully evaluated to draw meaningful conclusions.

## Gut-lung axis

**Figure 1.**
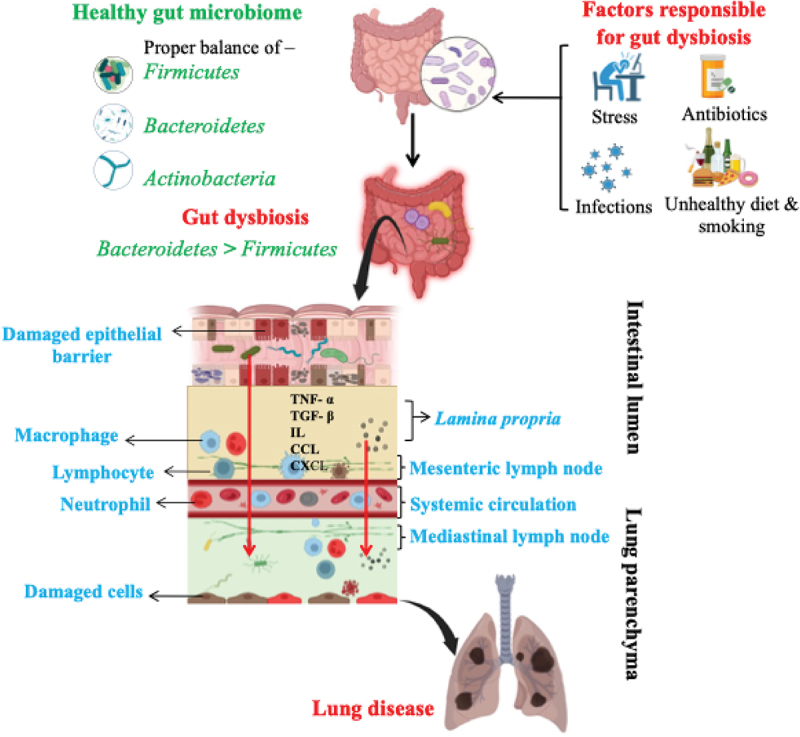
The illustration provides an overview of the gut-lung axis and its role in developing lung cancer. Factors such as antibiotics, stress, infections, smoking, and unhealthy diets contribute to gut dysbiosis, characterized by an imbalance in the *Firmicutes*/*Bacteroidetes* ratio within the gut microbiome. A healthy gut microbiome typically comprises *Firmicutes*, *Bacteroidetes*, and *Actinobacteria*, among others. Dysbiosis triggers an intense immune response, leading to the death of intestinal epithelial cells (IECs), disruption of tight junctions, and increased intestinal permeability. This “leaky gut” allows bacteria, microbial by-products, and inflammatory mediators – such as cytokines (TNF-α, TGF-β, IL-1β, IL-5, IL-6, IL-13, IL-17, IL-18, IL-33) and chemokines (IL-8, CCL2, CCL3, CCL4, CCL7, CCL20, CXCL5, CXCL8, CXCL10, RANTES) – to enter the bloodstream. These inflammatory molecules recruit immune cells like neutrophils and T cells, which cluster at the gut lining and subsequently enter systemic circulation, spreading inflammation to distant organs, including the lungs. Inflammatory signals can also travel from the gut to the lungs via the lymphatic system, activating alveolar macrophages and creating an inflammatory environment in the lungs. This inflammation damages alveolar epithelial cells (AECs), weakens the lung’s protective barrier, and contributes to lung conditions such as COPD, asthma, and lung cancer. Bacterial by-products absorbed through the gut wall also influence lung cell activity and immune responses. Illustration created with Biorender.com.

Abbreviations: TNF-α, Tumor necrosis factor-alpha; TGF-β, Transforming growth factor-beta; IL-1β, Interleukin-1 beta; IL, Interleukin; CCL, C-C motif chemokine ligand; CXCL, C-X-C motif chemokine ligand; RANTES, Regulated on activation, normal T cell expressed and secreted.

The lungs and gut have a bidirectional influence on health and disease, known as the gut-lung axis (GLA).^43^ Communication between the gut and lungs occurs via various mechanisms; one of the most commonly studied is gut dysbiosis, leading to lung disease, as shown in Figure 1. Various gastrointestinal tract (GIT) diseases, including inflammatory bowel diseases (IBD) such as ulcerative colitis (UC) and Crohn’s disease (CD), have been closely linked to respiratory conditions like chronic obstructive pulmonary disease (COPD), asthma, and cystic fibrosis. These conditions often overlap, highlighting a link between gut and lung health.^44,45^ For example, in a long-term study tracking patients for over 10 years, those with irritable bowel syndrome (IBS) were found to have a significantly higher chance of developing COPD. However, patients who received treatment for IBS had a notably lower risk of developing COPD.^46^ Researchers investigated the gut bacteria of asthmatic children. They discovered a favorable correlation between plasma metabolites (γ-tocopherol/β-tocopherol) and particular microbial species in the gut, like the *Christensenellaceae* family, and asthma-associated intestinal byproducts.^47^ Furthermore, *in-vivo* study in mice exhibited airway hyper-responsiveness and histologic indications of inflammation in the respiratory tract following treatment with a pure species of bacteria that is part of the human gut microbiome *Ruminococcus gnavus*.^48^ Various factors like stress, unhealthy diet, smoking, excessive use of antibiotics, and recurrent infections are responsible for gut dysbiosis, which influences the immune response and leads to chronic inflammation.^49^ For example, antibiotic-induced gut bacteria alteration in mice caused a reduction in lung expression of macrophage-inducible C-type lectin (Mincle), resulting in higher *Mycobacterium tuberculosis* (Mtb) survival. Notably, supplementation with *Lactobacillus* in mice restored Mincle expression on pulmonary dendritic cells and induced a concurrent anti-Mtb immune response.^50^ Furthermore, a cohort study accounting for potential confounders found that the incidence of lung cancer was 1.67 times higher in individuals with tuberculosis (TB) than in those without.^51^ TB is believed to increase lung cancer risk by inducing fibrosis and chronic inflammation, with inflammatory cells producing reactive oxygen and nitrogen species, prostaglandins, and cytokines. These substances and nitric oxide and ROS from *M. tuberculosis* can cause DNA damage and promote carcinogenesis.^52,53^ Additionally, antibiotics increase regulatory T cell (Treg) counts while reducing the prevalence of effector and memory T cells in lung tissue. This leads to suppressed immune responses, increased susceptibility to infections and impaired tumor immunity.^50^ *Firmicutes* and *Bacteroidetes* are the dominant bacterial phyla found in both the lungs and intestines of healthy humans. The F/B ratio is commonly used to diagnose gut dysbiosis. An increased *Bacteroidetes* and reduced *Firmicutes*, leading to a low F/B ratio, denote inflammation in the intestinal tissue, disruption of the intestinal barrier, erosion of the mucous layer, and increased permeability of the intestine. Elevation of microorganisms and their byproducts in the bloodstream due to a dysbiosis gut leads to an increase in inflammatory cytokines like TNF-α, TGF-β, IL-1β, IL-5, 6, 13, 17, 18, and 33, as well as chemokines like IL-8, CCL2, 3, 4, 7, 20, CXCL5, 8, and 10 in various tissues including the lung parenchyma.^54^ This stimulates neutrophils and T cells to enter the blood circulation and lymphatic system, through which the pro-inflammatory immune cells reach the lung parenchyma, causing alveolar barrier damage and stimulation of endothelial and immune cells within the lungs. The microbial metabolites reach the lungs through systemic circulation via gut mucosal tissue.^55^ Intestinal microbiota can prevent lung illnesses by influencing immunological homeostasis. Brown et al. reported the physiological benefits provided by the gut microbiota.^56^ Several gut microbiota species contribute to the production of interleukins, such as IL-17A, which is crucial for stimulating the production of granulocyte-macrophage-stimulating factors in the lungs. These factors help activate macrophages to eliminate potential threats in the lung alveoli.^57^ Additionally, lung microbiota alterations can lead to local and systemic immune changes. Such modifications may also enhance the intestinal immune response, as observed in *Staphylococcus aureus*-induced pneumonia, which can result in sepsis and apoptotic events in the gut.^58^ The gut microbiota interacts with the lungs through soluble microbial substances and bacterial metabolites that enter the bloodstream. These microbial components comprise pathogen-associated molecular patterns (PAMPs), including peptidoglycans and lipopolysaccharides.^59^ Beyond recognizing antigenic elements of the intestinal microbiota, the host detects microbiota-derived compounds, referred to as metabolites, which are absorbed through the intestinal lining and subsequently participate in pulmonary immunopathology and circulate via the bloodstream and lymphatic system. Short-chain fatty acids (SCFAs) such as propionic, butyric and acetic acids constitute the most common examples of metabolites obtained as a product of dietary fiber metabolism in the colon by gut bacteria. The SCFAs induce immune cell differentiation in peripheral circulation and remote organs like bone marrow. These cells are then transported to the lungs, thus regulating immunity and homeostasis of the lungs. In various cancer types, SCFAs, particularly butyrate, inhibit tumor growth and metastasis by modulating the cell cycle, autophagy, cancer-associated signaling pathways, and the metabolism of cancer cells. Furthermore, combining SCFAs with chemotherapy drugs has shown synergistic effects, enhanced the effectiveness of cancer treatments and reduced resistance to anticancer medications.^60^ Besides SCFAs, bacterial-derived histamine has also been shown to regulate lung immune responses. In a model of inflammatory airways, treatment with genetically modified *E. coli*, capable of producing histamine, significantly reduced lung eosinophilia and suppressed cytokine release in ovalbumin-sensitized mice.^61^ Probiotics and fecal microbiota transplantation (FMT) can potentially prevent and treat various lung conditions.^62,63^ Schuijt et al. demonstrated that microbiota-reduced C57BL/6 mice infected with *Streptococcus pneumoniae* experienced exacerbated bacterial spread, amplified inflammation, severe organ damage, and higher mortality rates than their microbiota-intact counterparts.^64^ FMT in mice with depleted gut microbiota restored lung bacterial loads and IL-10 and TNF-α levels. Specific SCFAs like butyrate and propionate facilitate a beneficial immune response by converting specific progenitor cells into specialized macrophages that help maintain lung health through anti-inflammatory actions and tissue repair.^65,66^ The gut-lung axis is a complex interaction that can influence lung health positively or negatively, depending on various factors related to immune activation and the state of the gut microbiome.^27^

## Gut dysbiosis in lung cancer: a comparison with healthy microbiome

**Figure 2.**
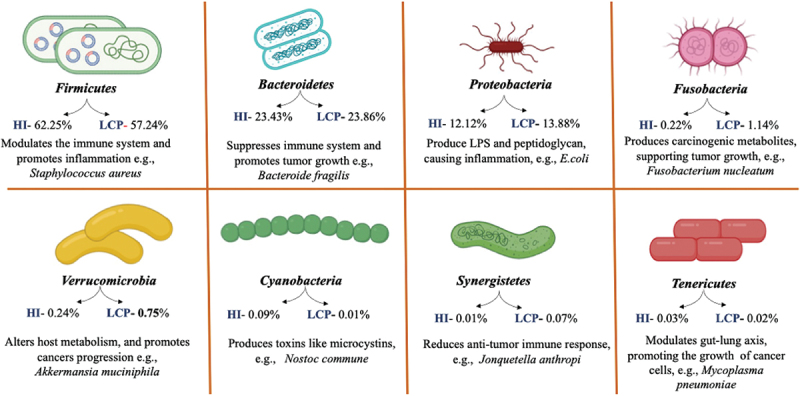
Phylum-level alterations in gut microbiota observed in lung cancer patients compared to healthy individuals. The illustration highlights significant shifts in microbial composition, including changes in the relative abundance of major phyla. These changes contribute to gut dysbiosis and may play a role in the progression of lung cancer through the gut-lung axis. Illustration created with Biorender.com.

Abbreviations: HI, Healthy individual; LCP, Lung cancer patient.

Alterations in the composition, function, or interaction of gut microbiota with the host have been linked to various diseases. Several studies have explored the relationship between gut microbiota and different types of tumors.^67,68^ Zhuang et al. found that higher amounts of *Enterococcus* in the gut microbiota are linked to lung cancer. In lung cancer patients, the overall decrease in gut microbiota function occurs, while *Enterococcus* and *Bifidobacterium* are the biomarkers for lung cancer.^69^ Compared to healthy individuals, it has been found that in patients with lung cancer, the amounts of *Kluyvera*, *Dialister*, *Escherichia-Shigella*, *Faecalibacterium* and *Enterobacter* are decreased. At the same time, levels of *Veillonella*, *Fusobacterium* and *Bacteroides* are markedly increased.^70^ The phylum-level alterations in gut microbiota observed in lung cancer patients compared to healthy individuals are shown in Figure 2. Zheng et al., in their cohort study, observed a marked change in the microbiota composition of patients with lung cancer compared to healthy people. They also observed distinct gut microbial profiles in patients with early-stage lung cancer. They concluded that levels of 3 phyla, 13 genera, and 20 species of microbes were reduced in lung cancer patients. In contrast, levels of 4 phyla, 11 genera, and 15 species were higher than those of healthy individuals. For example, phyla, such as *Firmicutes*, *Bacteroidetes*, *Proteobacteria*, and *Actinobacteria*, showed a notable distinction between the two groups. Specifically, the levels of phylum *Proteobacteria* and *Bacteroides* increased in lung cancer patients while decreased levels of species like *Firmicutes* and *Actinobacteria* were observed. These findings suggest lung cancer may be linked to increased pathogens and reduced specific probiotic microbes.^71^ Gui et al. observed in their study that non-small cell lung cancer (NSCLC) patients had dysbiosis of gut butyrate-producing bacteria. It includes a decrease in the number of *Faecalibacteriumprausnitzii*, *Clostridium leptum*, *Clostridial cluster I*, *Ruminococcus species*, *Clostridial cluster* XIVa and *Roseburia* spp., while causing no effect on the abundance of *Clostridial cluster* IV, and *Eubacterium rectal*.^72^ Liu et al. conducted a study involving 30 lung cancer patients, revealing a decreased abundance of gut microbiota and reduced microbial diversity. The findings highlighted a distinct pathogenic microbiome composition and probiotic genera’s notable depletion.^73^ Meanwhile, Botticelli et al. reported that levels of *Prevotella*, *Lactobacillus*, *Rikenellaceae*, *Streptococcus*, *Enterobacteriaceae*, *Oscillospira*, and *Bacteroides plebeius* in the stools of NSCLC patients were significantly higher than in healthy controls.^74^ Reports indicate that gram-negative bacteria, including *Haemophilus influenzae*, *Enterobacter* spp., and *Escherichia coli*, are also found in lung cancer cases.^75^ Chronic lung infections can trigger cancer development when changes in the microbiota create a hypoxic environment that promotes tumor growth. In lung cancer (LC), there is an observed rise in anaerobic respiration due to the preference for certain bacteria that thrive in tumor conditions. As LC advances, the population of these bacteria increases, further contributing to the hypoxic and pro-inflammatory environment around the tumor. Moreover, cancer treatments are also affecting the pathogenic microbiome.^76,77^ Understanding the mechanisms by which the microbiome influences lung cancer progression is crucial for improving patient survival and treatment responses. In contrast to the gastrointestinal tract, the lung microbiome is less well understood, and it is suggested that the relationship between the human microbiome and lung cancer is complex and multifaceted.^78^ Numerous preclinical and clinical studies have demonstrated that the gut microbiome can influence the host’s response to various cancer treatments, primarily through immune modulation.

Dysbiosis is both a consequence of treatment and a contributing factor to the differences in therapeutic responses observed among patients. Several studies have highlighted the significant role of the gut microbiome (GM) in lung cancer therapy, particularly in its influence on immune checkpoint inhibitor (ICI) therapy. The GM affects the differentiation of regulatory T cells, leading to alterations in immune-modulation mechanisms, as evidenced by direct assessments of drug metabolism and the host’s immune response. Furthermore, the GM may enhance the effectiveness of chemotherapy in lung cancer.^79^ The specific composition of gut microbes can influence the effectiveness of various conventional chemotherapy agents, as demonstrated in mouse model studies. For example, cyclophosphamide (CP), a standard chemotherapy drug, reduces the height of intestinal villi and disrupts the intestinal barrier, leading to the movement of commensal bacteria into secondary lymphoid organs and an increase in inflammatory cells. Research has also shown that antibiotics targeting gram-positive bacteria, unlike those targeting gram-negative bacteria, significantly decrease the efficacy of CP. Certain gram-positive bacteria, such as *Lactobacillus johnsonii*, *Lactobacillus murinus, Enterococcus hirae*, and segmented filamentous bacteria, enhance CP’s antitumor effects.^80,81^ In patients with advanced lung and ovarian cancer exhibiting elevated levels of *E. hirae* and *Barnesiella intestinihominis*, specific Th1 cell memory responses were associated with prolonged progression-free survival. Based on this finding, it has been proposed to include certain *Enterococcus* and *Barnesiella* species in a tailored microbiota cocktail administered alongside CP and other alkylating agents. In the future, these bacteria or their specific immuno-modulatory products and metabolites could be used as adjuvants to enhance the effectiveness of current chemotherapy treatments.^80^ In lung cancer patients undergoing treatment with immune checkpoint inhibitors, the gut microbiome of those who respond to therapy differs significantly from that of non-responders. Routy et al. found that a higher abundance of *Akkermansia muciniphila* was significantly associated with better responses to anti-PD1 therapy in lung cancer patients. Additionally, other research indicated that *A. muciniphila* is linked to positive outcomes with immune checkpoint inhibitor treatment. Supplementing with this species enhanced responses to treatment, while an abnormal gut microbiota composition is related to resistance against immune checkpoint inhibitors.^82^

**Figure 3.**
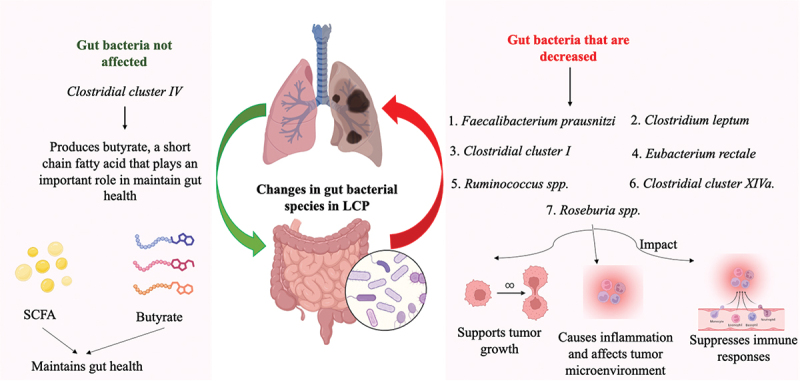
The illustration depicts species-level alterations in gut microbiota composition observed in lung cancer patients (LCP). Dysbiosis in the gut microbiome is characterized by changes in the relative abundance of specific bacterial species, potentially contributing to lung cancer progression. Altered microbial communities influence systemic inflammation, immune modulation, and metabolic pathways, further impacting the gut-lung axis. Illustration created with Biorender.com.

Abbreviations: LCP, Lung cancer patient.

Additionally, some chemotherapeutics, like Doxorubicin, exhibit similar effects to Irinotecan and can result in adverse reactions in both the gastrointestinal and respiratory systems. Consequently, targeting the microbiota is proposed as a strategy to reduce the toxicity of various chemotherapy agents.^83,84^ Research indicates that a high intake of yogurt can significantly lower lung cancer risk by 30%, suggesting that prebiotics and probiotics may offer protective effects against lung carcinogenesis. Additionally, changes in gut diversity could serve as a potential biomarker for diagnosing and treating lung cancer. However, the role of the gut microbiome in the development and progression of lung cancer requires more investigation. Moreover, the microbiome’s potential to effectively modulate anticancer treatments should also be further studied and assessed.^19,85^ The species-level alterations in gut microbiota composition observed in lung cancer patients (LCP) are explained in Figure 3.

## Mechanisms of gut microbiota causing lung cancer

According to research, it has been observed that there is a link between microbial influence on carcinogenesis within tumors and lung cancer progression. Overall, six key processes have been identified as putative modes of action. In particular, these six pathways interact and influence one another during carcinogenesis.^86^

### Immune reactions influenced by the microbiome

The gut microbiota is essential for maintaining a healthy balance in our lung immune system. It helps shape how our body responds to immune challenges by regulating innate and adaptive immune responses. The balance between lung immune response and immune regulation is disturbed by gut dysbiosis, which results in either increased immune response or impaired immune vigilance function that leads to cancer-promoting inflammation or reduced antitumor effectiveness, respectively. This provides a suitable environment for developing lung cancer.^27^ Since the gut microbiome can influence a host’s immunological functions, a thorough understanding of the immune response and the inflammatory pathways influenced by bacteria is required.^86^ The basic mechanism in pulmonary immune function involves the activation of immunity in the intestine by intestinal flora and the migration of the activated immune cells to the lungs. After activation, immune cells, especially effector T cells and innate immune cells such as neutrophils, leave the gut-associated lymphoid tissue (GALT) and travel into the bloodstream. This process is guided by distinct chemokines and adhesion molecules. Chemokine-mediated targeting of lymphocytes is believed to be the cause of lung-targeted movement of lymphocytes.^27^ Research by Samir and colleagues found that antibiotics can upset the balance of gut bacteria, a condition known as dysbiosis. This disruption can speed up the progression of cancers such as B16-F10 melanoma and lung cancer in mice. One reason for this faster progression is a drop in TNF-α levels, which affects the expression of adhesion molecules like intercellular adhesion molecule-1 (ICAM-1). As a result, immune cells, or leukocytes, have a more challenging time reaching the tumors, leading to fewer activated CD8+ T cells in the tumor environment.^87^ Additionally, Rodrigue and colleagues discovered that an imbalance in gut bacteria, known as dysbiosis, can significantly weaken the immune response in the lungs. This leads to a decrease in several types of immune cells, including macrophages and natural killer (NK) cells. Additionally, this dysbiosis lowers the levels of Fms-like tyrosine kinase 3 (Flt3)-ligand, a key cytokine involved in immune cell development. It hampers the formation of dendritic cells from their bone marrow precursors.^88^ Negi and her team found that when gut microbiota is out of balance, it reduces levels of specific proteins in lung macrophages. They also found that antibiotics can decrease the number of effector and memory T cells essential for a strong immune response.^50^ Overall, these studies highlight the critical role of gut microbiota in regulating immune responses and cancer progression. Another study found that innate intestinal lymphocytes are essential in keeping the lungs healthy. They discovered that the gut microbiota helps transport these cells to the lungs by boosting the expression of the C-C chemokine receptor type 4 (CCR4) homing receptor in group 3 innate lymphoid cells (ILC2). Interestingly, this process is not linked to the growth or death of innate lymphoid cells ILC3s.^89^ CCR4 and its ligand CCL17 facilitate T-cell migration to the lungs, while CD11c+, CD8+ cells link gut microbiota antigens to lung immunity via toll-like receptor 4 (TLR4).^89,90^ Researchers have found that a lack of IL-17C enhances the growth and spread of cancer cells.^91^ Ngo et al., in their study, found that when bacteria activate MYD88 in myeloid cells, it leads to the signaling of IL-23. This process can promote tumor growth and the development of IL-17 responses within tumors. In simpler terms, MYD88 helps send signals to make tumors grow faster and trigger specific immune responses that may support cancer progression.^92^

In addition to chemokine receptors, segmented filamentous bacteria (SFB) have been found to stimulate the production of pulmonary auto-antibodies. The T helper type 17 (Th17) cells are essential for the lung-related issues associated with SFB. Intestinal Th17 cells induced by SFB preferentially migrate to the lungs, guided by CCL20, which also attracts them to inflammatory sites in the CNS.^93–95^ SFB also promotes the selective expansion of Th17 cells expressing dual T cell receptors, enhancing autoimmune inflammation by recognizing self-antigens and SFB epitopes in various peripheral tissues.^93^ In a recent study on how the gut microbiome affects immune protection in lung cancer, it was found that enterotoxigenic *Bacteroides fragilis* (ETBF) can activate the signal transducer and activator of transcription 3 (STAT3) pathway through a specific Th17 immune response in mice, suggesting that certain human gut bacteria might promote cancer via this Th17-dependent mechanism. Li et al. found that gut microbiome disruption allows tumor-secreted proteins, such as cathepsin, to bind to TLR4, triggering M2 polarization of tumor-associated macrophages (TAMs) via an mTOR-dependent pathway. This process enhances the invasion and metastasis of non-small cell lung cancer (NSCLC) cells through the NF-κB pathway.^96,97^ Harmful bacteria, like those causing influenza, boost the production of IL-17C in the epithelial cells of patients with COPD. This increase contributes to tumor growth by promoting neutrophilic inflammation within the tumor microenvironment.^91^ Additionally, bacteria in the lungs can affect the expression of innate immunity genes such as IL-5, 10, and IFN. In fact, the levels of PD-L1 on CD11b+ dendritic cells and Foxp3+ CD25+ regulatory T cells were higher in the lungs of specific pathogen-free neonates than in germ-free (GF) mice.^98^ Moreover, commensal bacteria may alter immune responses in the airway mucosa through inflammasomes and provide signals for immunological activation following influenza virus infections.^99^ Researchers have found in preclinical models that changing the gut microbiome can influence the host’s immune responses and their vulnerability to lung infections.^99,100^ For instance, GF mice, which lack gut flora, displayed significant immune system issues, such as a thin mucus layer, abnormal immunoglobulin production, and a decrease in both the size and number of lymph nodes.^101^ Dzutsev et al. proposed that lung cancer patients with low levels of certain bacteria might not respond well to immunotherapy. This could be partly due to changes in their bacterial makeup, which play a key role in shaping the immune system.^102^ Researchers found that the body’s response to bacteria can sometimes lead to too much cell growth, increasing cancer risk. For example, Elangovan et al. found that activating inflammatory pathways, like those involving microbe-associated molecular patterns (MAMPs) and pattern recognition receptors (PRRs), can encourage epithelial cells to grow and survive in certain situations. This process can ultimately contribute to the development of cancer.^103^ In contrast, some research indicates that TLRs, activated by bacteria, might help combat cancer in certain organs, like the colon, stomach, liver, and pancreas. Instead of promoting cancer, these receptors can trigger immune responses that may inhibit tumor growth or help the body fight off cancer. While some inflammatory pathways can lead to cancer, TLRs might play a protective role in these specific areas by responding positively to bacterial presence.^92^ Additionally, studies reveal that nucleotide-binding oligomerization domain-like receptors (NOD) are pattern recognition receptor (PRR) in cell membranes. NOD1 plays a protective role by acting as a barrier that prevents inflammation from leading to cancer. Meanwhile, NOD2 helps regulate bacteria in the body and lowers the risk of colorectal cancer (CRC). Studies with mice that don’t have these NOD receptors show just how crucial they are for keeping a healthy balance of gut bacteria and reducing cancer risk. Overall, this suggests that different immune pathways influenced by bacteria are really important for our health and can affect disease development.^104,105^

### Bacterial influence on inflammatory pathways

Intestinal bacteria influence inflammatory responses in the gut and contribute to chronic lung inflammation through a connection known as the gut-lung axis.^106^ An imbalance in gut bacteria, known as gut dysbiosis, can harm the intestinal barrier and make it more permeable. This means that the barrier becomes less effective at protecting the body, allowing unwanted substances to pass through more easily.^107,108^ When pathogenic bacteria invade during the first infection, they cause inflammation and produce more mucus. This inflammation can damage the respiratory lining, which weakens the body’s natural immune response. As a result, the lungs become more vulnerable to further infections from harmful germs, creating a harmful cycle of ongoing issues.^106^ Therefore, an imbalance in gut microbes and their byproducts may trigger chronic inflammation, contributing to the onset and progression of lung cancer. A recent study revealed that polymeric immunoglobulin receptor deficiency impairs mucosal immunity, enabling pathogen invasion, inflammation, and bacterial lung infections. In chronic inflammation, gammaproteobacteria outcompete other microbes by utilizing inflammatory metabolites and reactive nitrogen species (RNS), perpetuating inflammation and potentially accelerating lung cancer development.^109,110^ Microorganisms and their byproducts that penetrate the intestinal mucosa trigger TLRs, producing inflammatory mediators and factors. These then play a role in the inflammatory processes in the lungs through the lymphatic system and bloodstream. Another study reported that gut dysbiosis, marked by a notable rise in *Enterobacteriacea*e, activates TLR4 in the intestine. This triggers inflammation, raises IL-1β levels in the bloodstream, transmits inflammatory signals to the lungs, and activates the NF-κB pathway, ultimately resulting in lung inflammation.^111^ Likewise, Jia and colleagues found that intestinal microbiota dysbiosis can influence the TLR4/NF-κB signaling pathway in pulmonary immunity. This alteration leads to oxidative stress and inflammation, which play a role in lung pathology by affecting the intestinal barrier.^112^ Research has shown that *Enterobacter* and *Escherichia-Shigella* are strongly linked to elevated serum neutrophil-to-lymphocyte ratios (NLR). At the same time, Dialister is associated with lower NLR and platelet-to-lymphocyte ratio levels. Additionally, serum levels of CTLA-4 and IL-12 were found to be correlated with Dialister.^70^ Another study noted that the presence of *Enterococcus* and *Helicobacter* genera was closely related to IL-6 levels.^113^ Increased levels of *Lachnospiraceae* and *Ruminococcaceae* in fecal microbiota were linked to higher lung concentrations of TNF-α and IL-17.^114^ Furthermore, antibiotic treatment in mice significantly reduced both bacterial population and diversity, along with a notable increase in IL-6 levels in bronchoalveolar lavage fluid (BALF).^115^ Besides gut microbiota, the metabolites produced by gut microbiota also can potentially affect the balance of pulmonary inflammation. Recent studies have found that certain gut bacteria can produce metabolites with pro-inflammatory effects, including 12, 13-diHOME and bile acids. When mice were given 12, 13-diHOME, they showed increased pulmonary inflammation and reduced regulatory T cells (Treg) in their lungs, making them more susceptible to asthma due to impaired immune tolerance. Additionally, 12, 13-diHOME altered the expression of PPARγ-regulated genes in human dendritic cells, leading to lower secretion of anti-inflammatory cytokines and fewer Treg cells *in-vitro*. Moreover, higher concentrations of bile acids produced by gut microbiota were significantly linked to increased levels of inflammatory markers such as IL-1β, 6, and 8 in the BALF of cystic fibrosis patients.^116,117^ SCFAs also play a vital role in regulating inflammation. It has been observed that there is a decrease in SCFA-producing bacteria and overall serum SCFA levels in lung cancer patients, which shows a correlation between the metabolite and inflammation regulation. Overall, the gut microbiota plays an active role in lung inflammation, both directly and through its metabolites, by influencing microbial-cytokine interactions in the gut and throughout the body. This increases pro-inflammatory effects, diminished anti-inflammatory responses, and chronic systemic inflammation, creating an environment conducive to lung cancer development. Consequently, regulating gut flora could offer a new and promising approach to maintaining host balance and preventing lung cancer.^27^ Many researchers believe that the inflammation caused by bacteria might work similarly to immune responses, but the specific mechanisms that lead to cancer are still unclear compared to immune reactions. Factors like regulatory T-cell subsets, TLRs, inflammatory cytokines, surfactant protein D, and others have been suggested as possible contributors. However, bacterial lung infections can often be detected earlier because we can easily measure inflammatory markers in the blood, such as C-reactive protein (CRP), TNF-α, and IL-6. Notably, higher levels of CRP are linked to the types of gut bacteria found in lung cancer patients. This suggests that increased inflammatory markers in the blood could indicate a risk for bacterial imbalances related to lung cancer.^118,119^

### The interaction between host metabolism and bacterial metabolites

Metabolites produced by gut bacteria can enter the bloodstream and affect the health and functioning of distant organs, like the lungs.^59^ Changes in gut bacteria can produce metabolites that interfere with the body’s natural ability to suppress tumors. These metabolites can fuel cells, boost the production of essential molecules, or disrupt important signaling proteins, upsetting the body’s metabolic balance and encouraging tumor growth.^120^ Certain metabolites can suppress the immune system and heighten inflammation, promoting cancer. Analyzing the metabolome may aid in early cancer detection and diagnosis.^86^ Bacterial metabolites influence immune responses by affecting the differentiation of T-cells like effector T cells and Tregs or by releasing Th17 and triggering inflammation, which may promote tumor formation.^121^ Additionally, it has been found that certain bacterial metabolites are linked to lung cancer development. Cytolethal distending toxin (CDT) from gram-negative bacteria like *Actinobacillus* induces apoptosis in lung adenocarcinoma cells. At the same time, *Granulicatella adiacens* is linked to lung cancer and affects polyamine metabolism, with elevated polyamines associated with cancer progression.^122,123^ Disruptions in gut microbiome homeostasis can generate carcinogenic metabolites, like acetaldehyde and deoxycholic acid (DCA), which have been linked to esophageal and liver cancers. Another bacterial byproduct, lipopolysaccharides (LPS), can worsen asthma by making the lungs more sensitive to allergens.^124,125^ Research by Bei et al. showed that certain bacteria and their metabolites play a role in other diseases, like *Pseudomonas aeruginosa* and *Rothia mucilaginosa*, which may contribute to the progression of cystic fibrosis.^126^ Additionally, bacterial metabolites affect important metabolic pathways, like glycerophospholipid and lineolate pathways, which are involved in pneumonia in people with HIV.^127^ Cyanobacteria abundance is higher in lung adenocarcinoma, producing toxins like microcystin that increase PARP1 expression, promoting inflammation and cancer. This was confirmed in microcystin-treated A427 lung cancer cells. This indicates a potential link between certain bacteria, inflammation, and cancer growth, highlighting the role of microbial toxins in tumor development.^128^ *Bacteroidaceae* and *Prevotellaceae* can hydrolyze red ginseng ginsenosides, enhancing their anticancer effects against lung cancer, while reduced symbiotic bacteria may worsen cancer progression.^129^ The SCFAs are key metabolites produced by beneficial gut bacteria.^65^ Recent research shows that dietary fiber helps gut bacteria produce more SCFAs through fermentation.^130^ Trompette et al. found in their study that dietary fiber, such as inulin, boosts SCFA production by altering gut bacteria. This improves immune responses, like in mice fighting the flu, by reducing damage caused by neutrophils and enhancing the antiviral activity of CD8+ T-cells.^65^ SCFAs also have anti-inflammatory properties that lower the risk of colon and breast cancer. On the downside, harmful metabolites like acetaldehyde are associated with cancer,^131^ and DCA, produced in people with obesity, increases the risk of liver cancer.^132^ Interestingly, DCA can also help maintain gut health by influencing cellular repair mechanisms by activating histone deacetylase-like 3 (HDAC3), a protein involved in gene regulation, through inositol triphosphate signaling.^86^ The findings suggest that understanding bacterial metabolites and their drug interactions may provide new avenues for lung cancer treatment. The important bacterial metabolites, their sources, and their role in lung cancer progression are summarized in a tabular format in Table 1.

**Table 1.** Gut microbiota metabolites, their microbial sources, and their impact on lung cancer progression.

Sl. No	Metabolites	Microbial source	Impact on lung cancer progression	References
1	SCFAs (Butyrate and Propionate)	*Lactobacillaceae*, *Ruminococcaceae, Lachnospiraceae.*	Inhibit tumor growth and metastasis by modulating the cell cycle, autophagy, cancer-associated signaling pathways, metabolism of cancer cells, anti-inflammatory actions and tissue repair.	^60,65,66,133,134^
2	Secondary bile acids	*Bacteroides*, *Clostridium*, *Bifidobacterium,Lactobacillus, Listeria*	Increased levels of inflammatory markers such as IL-1β, 6, and 8 in the BALF of cystic fibrosis patients.	^116,117,135^
3	LPS	Gram-negative bacteria	Worsen asthma by making the lungs more sensitive to allergens and activate TLR4 receptors to produce inflammation-related genes.	^124,125,136^
4	12,13-diHOME	*Enterococcus faecalis, Streptococcus, Bifidobacterium bifidum* and*Lactobacillus*	Increased pulmonary inflammation, reduced Treg cells, and impaired immune tolerance by altering PPARγ-regulated gene expression in dendritic cells, leading to fewer anti-inflammatory cytokines *in-vitro*.	^116,116,117^
5	Cytolethaldistending toxin (CDT)	*Actinobacillus*	Induces apoptosis in lung adenocarcinoma cells.	.^122,123^
6	Lithocholic acid	*Bacteroides, Bifidobacterium*, *Clostridium, Lactobacillus,Listeria*, and *Enterococcus*.	Damages DNA and promote cancer development.	^86,137,138^

Abbreviations: SCFAs, Short chain fatty acids; IL, Interleukin; BALF, Bronchoalveolar lavage fluid; LPS, Lipopolysaccharides; TLR, Toll-like receptor; 12,13-diHOME, 12,13-dihydroxy-9Z-octadecenoic acid; PPARγ, Peroxisome proliferator-activated receptor gamma; CDT, Cytolethal distending toxin; DNA Deoxyribonucleic acid.

### Imbalance in the microbiome (dysbiosis)

When the microbiome becomes unbalanced (dysbiosis), beneficial bacteria decrease, and harmful, inflammation-causing bacteria increase. This imbalance can contribute to cancer development in several ways. In the case of lung cancer, it’s believed that the cancer is driven more by this microbial imbalance rather than by any single harmful pathogen.^139^ Dysbiosis has been associated with higher levels of harmful substances, like genotoxins and metabolites, that can trigger mutations and cancer, along with disrupting the immune system. For instance, an experiment with mice exposed to radiation showed that an imbalanced gut microbiome made the mice less able to tolerate DNA damage, highlighting the impact of dysbiosis on the body’s defenses.^140,141^ Studies have shown that changing the gut bacteria in mice can impact how well cancer treatments, like immunotherapy and chemotherapy, work. The connection between the gut and lungs (the gut – lung axis) plays a key role in shaping immune responses in the lungs. Short-chain fatty acids (SCFAs) from gut bacteria can inhibit certain enzymes (HDACs) involved in immune regulation. When the gut – lung balance is disrupted (dysbiosis), it can weaken the body’s ability to fight tumors and shift conditions to favor lung cancer development.^43,142^ Factors like inflammation, changes in diet, infections, and a lack of the nucleotide-binding oligomerization domain-containing protein 2 gene (NOD2 gene) can all disrupt the balance of the microbiome, leading to dysbiosis.^143^ Researchers analyzed lung cancer patients’ bronchoscopic samples and found higher levels of certain gram-negative bacteria, like *Haemophilus influenzae*, *Enterobacter spp*., and *E. coli*.^75^ This suggests that imbalances in bacterial populations (dysbiosis) can spread throughout the body. Sobhani et al. found that changes in gene methylation might explain the link between dysbiosis and cancer.^144^ While they observed connections between dysbiosis, tissue changes, and DNA patterns in animals, it remains unclear whether dysbiosis and inflammation play a direct role in the early stages of cancer.^145^

### Effects of genotoxicity and virulence

Disrupted microbiome balance can lead to toxin production and free radical formation, promoting cancer development. Aging and harmful exposures further aggravate this imbalance, fostering toxins or antimicrobial agents that harm competing bacteria.^146^ Studies have shown that harmful and commensal microorganisms can mutate the host organism’s cells. Three notable genotoxins that can directly harm the DNA of host cells are typhoid toxin from *Salmonella enterica* serovar Typhimurium, cytotoxic distension toxin (CDT) produced by several gram-negative bacteria like *Helicobacter*, *Escherichia coli*, *Shigella dysenteriae*, *Haemophilusducreyi*, and *Campylobacter jejuni*, and colibactin from specific strains of *Escherichia coli* in the B2 phylogenetic group. When these toxins enter the target cell’s nucleus, they can cause DNA breaks and initiate a DNA damage response (DDR). This response can halt the cell cycle or lead to cell death, depending on the type of damage inflicted and the toxin concentration.^147,148^ Recent research indicates that gut bacteria can produce enzymes known as the Estrobolome, which help metabolize estrogen. This discovery raises new questions about how these bacteria affect the balance of estrogen and testosterone in the host, a balance that plays a crucial role in various cancer processes.^149^ Additionally, past studies have identified ROS as key players in DNA damage responses. Bacterial dysbiosis can alter the levels of ROS, leading to DNA damage and potentially contributing to cancer development. The lungs are the primary organs responsible for gas exchange in the body, making them more vulnerable to ROS exposure due to various lifestyle factors such as smoking, occupational hazards, and prolonged exposure to pollutants and allergens. DNA adduct formation induced by ROS contributes to intrinsic cancer risk. Additionally, ROS produced by inflammatory cells can activate oncogenes like Jun and Fos, with elevated Jun expression being directly associated with the development of lung cancer.^150–152^ Additionally, oxidants like ROS trigger the activation of transcription factors like nuclear factor kappa B (NF-κB), activator protein 1 (AP-1), mitogen-activated protein kinases (MAPKs), phosphoinositide kinase (PI3K), and activated serine-threonine kinase (AKT), leading to histone acetylation and deacetylation. These processes play a role in carcinogenesis induced by oxidants. The likely mechanisms of ROS-induced carcinogenesis involve DNA damage, inhibition of apoptosis, activation of cell signaling pathways, including AP-1 and NF-κB, disruption of tumor suppressor gene p53, and the modulation of cellular defense systems.^153^ Microbiome modulators such as probiotics and synbiotics have been shown to exhibit antioxidant effects.In a study by Aghamohammad and colleagues, probiotics like *Lactobacillus* spp., *Bifidobacterium* spp. and their mixture was found to reduce the expression of janus kinase (JAK) genes and the toll/interleukin-1 receptor domain-containing adaptor protein(TIRAP), interleukin-1 receptor-associated kinase 4 (IRAK4), nuclear factor-kappa B essential modulator(NEMO), and receptor-interacting protein(RIP) genes in the NF-κB pathway compared to cells treated with sonicated pathogens. Hence, probiotics such as *Lactobacillus* spp. and *Bifidobacterium* spp., when used as nutritional supplements, may help alleviate inflammation-related diseases.^154^ Lactic acid bacteria (LAB) are extensively utilized as probiotics in fermented foods, beverages, and food supplements for both humans and animals due to their wide range of health benefits, many of which are attributed to their antioxidant properties.^155^ A significant contributor to ROS production in the body is nicotinamide adenine dinucleotide phosphate oxidase (NADPH oxidase). Research involving *Lactobacillus* strains has demonstrated that it can effectively reduce NADPH oxidase (NOX) activity and decrease the expression of NOX-1 and NOX-4 mRNA in spontaneously hypertensive rats, suggesting a potential role in mitigating ROS-related damage.^156^ Cyclo-oxygenase 2 (COX-2), a key enzyme associated with ROS production, shows a reciprocal relationship with ROS.^157^ Notably, pretreatment with *Lactobacillus acidophilus* has been shown to significantly downregulate COX-2 expression in bovine thymic macrophages challenged with the pathogenic bacterium *Aeromonas hydrophila*, further supporting the antioxidant capacity of *Lactobacillus*^158^ Additionally, cytochrome P450 (CYP), which functions as the terminal oxidase in the electron transport chain, is another major source of continuous ROS production.^159^ In this regard, *Lactobacillus casei* has been reported to reduce the expression of CYP1A1 in various sections of the jejunum, colon, ileum, and cecum in male rats, further underscoring the potential of LAB to modulate ROS-producing enzymes.^160^ In a randomized controlled clinical trial (RCT) conducted by Zolghadrpour et al., the effects of a newly developed synbiotic yogurt on oxidative stress in adults with metabolic syndrome (MetS) were examined. The study found that daily consumption of synbiotic yogurt resulted in a statistically significant improvement in glutathione peroxidase levels and total oxidant status compared to regular yogurt. Additionally, total antioxidant capacity and superoxide dismutase levels showed significant increases in the intervention group compared to baseline levels. The trial concluded that daily intake of synbiotic yogurt, containing native strains of *Lactobacillus plantarum*, *Lactobacillus pentosus*, and *Chloromyces marcosianus* yeast over 12 weeks, was associated with notable improvements in oxidative stress status in adults with MetS.^161^ These findings highlight the ability of probiotics and synbiotics to modulate key enzymes involved in ROS production, suggesting their potential role in oxidative stress reduction. Certain bacterial toxins, including CDT,^122^ cytotoxic necrotizing factor 1(CNF1),^162^ and the toxin from *Bacteroides fragilis*, are known to cause double-stranded DNA damage.^163^ Furthermore, hydrogen sulfide and superoxide radicals bacteria produce have been linked to chromosomal instability.^164^ Additionally, a product called Fad A, produced by *Fusobacterium nucleatum*, has been shown to influence catenin signaling by interacting with E-cadherin.^165^ Certain bacteria in the lungs can transfer plasmids containing genetic traits to tumor cells through exosomes, leading to cancer-related functions like drug resistance and inflammation. Additionally, secondary metabolites like deoxycholic and lithocholic acid, produced by gut bacteria from bile acids, can damage DNA and promote cancer development. The creation of these harmful metabolites in the lungs due to metabolic imbalances may play a role in lung cancer progression.^86^ Research shows that radiotherapy in lung cancer patients not only damages normal lung cells and bacteria but also leads to radiation-induced toxicity. This increased vulnerability can make patients more susceptible to infections from bacteria like *Escherichia coli*, *Staphylococcus aureus*, *Pseudomonas aeruginosa*, and *Staphylococcus epidermidis*. However, promising findings from Chen et al. suggest that fecal microbiota transplantation (FMT) could mitigate radiation damage, reduce oxidative stress, and enhance lung function in mouse models. High levels of beneficial gut bacteria can help protect healthy lung cells by activating MAPK/NF-κB signaling pathways and promoting the secretion of prostaglandin F2α (PGF2α), which inhibits cell death.^166^

### Bacteria-induced epigenetic alterations

**Figure 4.**
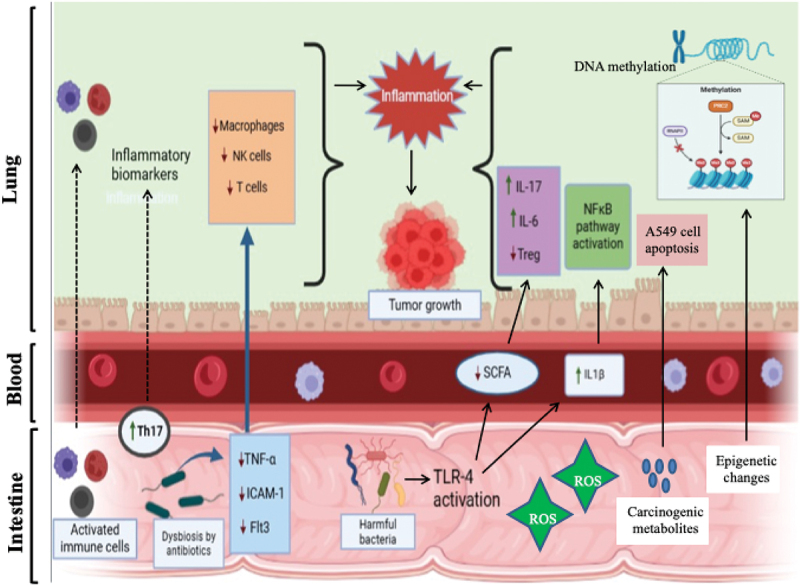
Mechanisms of gut microbiota causing lung cancer. The figure represents various mechanisms through which gut dysbiosis can lead to the development of lung cancer. The pathways involved are immune reactions influenced by the microbiome, bacterial influence on inflammatory pathways, the interaction between host metabolism and bacterial metabolites, imbalance in the microbiome (dysbiosis), effects of genotoxicity and virulence, and bacteria-induced epigenetic alterations. These interconnected mechanisms demonstrate the critical role of the gut microbiome in systemic inflammation, immune modulation, and lung cancer pathogenesis. Illustration created with Biorender.com.

Abbreviations: NK cells, Natural killer cells; TNF-α, Tumor necrosis factor-alpha; ICAM-1, Intercellular adhesion molecule-1; FLT3, fms-like tyrosine kinase 3; Th17, T helper 17; IL, Interleukin; NF-κB, Nuclear factor kappa-B; ROS, Reactive oxygen species; TLR-4, Toll-like receptor 4; SCFA, Short chain fatty acids.

Lung cancer involves disruptions in epigenetic processes, which, combined with genetic variations, play a key role in its development and progression.^167^ The lung’s constant exposure to environmental pollutants and bacteria makes it particularly vulnerable to changes in gene regulation. Influenced by the surrounding microenvironment, these epigenetic shifts can disrupt normal cellular functions, increasing the risk of cancer development.^168^ Epigenetic control of gene expression occurs at three levels: DNA methylation, chromatin modification, and non-coding RNAs. DNA methylation is especially important for turning off genes and can reshape the structure of chromatin.^119,169^ DNA hyper-methylation, which can silence key tumor suppressor genes, may play a role early in the development of lung cancer. Research suggests that the microbiome helps regulate these epigenetic changes. Growing evidence points to a link between microbiome imbalances and gene regulation disruptions, contributing to lung cancer progression.^170^ The surrounding tissue environment also plays a key role in balancing beneficial bacteria while staying alert to invading pathogens to trigger the right immune response.^171,172^ In the case of lung cancer, the spread of opportunistic pathogens can disrupt the enzymes and factors responsible for maintaining chromatin structure. Studies show that bacteria and their metabolites can modify the host’s epigenetic processes, helping the microbes survive, replicate, and evade the body’s natural immune defenses.^173^ When bacterial lipopolysaccharides (LPS) activate the TLR4 receptor, it sends NF-κB into the nucleus, kick-starting the production of inflammation-related genes. Some genes, known as early response genes, are ready to be activated quickly due to their epigenetic and transcriptional readiness. In contrast, activating late response genes requires additional signals and changes to chromatin structure. Both helpful and harmful bacteria can alter the chromatin landscape, influencing inflammation and other cellular functions to establish themselves and thrive within the host.^171,172^ A review highlights how bacteria can alter various non-coding RNAs, which play a role in reshaping chromatin structure.^174^ Relatedly, Hu et al. explored the potential link between oncogenic viruses – such as HPV, Merkel cell polyomavirus (MCPyV), Epstein – Barr virus (EBV), Jaagsiekte sheep retrovirus (JSRV), and John Cunningham virus (JCV) – and lung cancer.^175^ It is evident that viruses can manipulate the host’s chromatin structure to support their survival and spread.^176,177^ Thus, further research on the molecular mechanisms behind these virus-driven epigenetic changes and their contribution to lung cancer development is necessary. Besides viruses, different metabolites produced by microorganisms, such as butyrate, folate, and biotin, have the ability to alter epigenetic processes.^178^ For instance, lung bacteria that produce biotin are essential for maintaining biotinylated proteins, including specific histones (H3, H4, and H2A). This biotinylation is crucial in regulating DNA repair, gene silencing, and cell growth.^179^ Rossi et al. reviewed that folate, produced by various bacteria like *Lactobacillus* and *Bifidobacteria* plays a key role in generating 6-methyltetrahydrofolate. This compound acts as a methyl group donor, directly influencing DNA methylation and gene regulation.^180^ Wu et al. found that the microbial metabolite phytate activates HDAC3 through inositol trisphosphate, supporting intestinal balance and tissue repair.^181^ Butyrate is a short-chain fatty acid produced through the microbial fermentation of dietary fibers in the colon. Over the past 10 years, numerous beneficial effects of butyrate have been shown at both the intestinal and extraintestinal levels. Butyrate exerts its effects through various mechanisms, many of which involve epigenetic regulation of gene expression by inhibiting histone deacetylase.^182^ Epigenetics explores the mechanisms that shape chromatin structures and stably regulate gene expression, thereby determining cell identity and function and enabling cells to adapt to their environment without altering the DNA sequence. Three main epigenetic mechanisms – histone acetylation, DNA methylation, and non-coding microRNAs – play a key role in modifying genes’ expression in both physiological and pathological processes.^183^ Histone acetylation is thought to increase a gene’s accessibility to transcription machinery, while deacetylated histone tails, being highly charged, are believed to bind tightly to the DNA backbone, restricting the access of transcription factors to the genes. Environmental factors, such as dietary compounds, can modulate histone acetylation and deacetylation, potentially preventing diseases and promoting health. Recently, there has been growing interest in dietary histone deacetylase inhibitors (HDACi), especially butyrate, due to their influence on epigenetic mechanisms, offering promising prospects for more targeted and effective therapeutic strategies in the prevention and treatment of various diseases.^184^ Metabolites produced by microbes can enter the bloodstream and influence the immune environment in lung cancer. Oncometabolites contribute to lung cancer development by damaging the host’s DNA, while tumor-suppressing metabolites help regulate the immune system to fight against the aggressive nature of cancer cells. These metabolites could even have significant potential in enhancing the efficacy of immunotherapy for lung cancer. Exploring microbial metabolites found in patient stool or blood samples, given the communication between the gut and lungs, can provide valuable insights for diagnosing and selecting lung cancer therapies. Additionally, targeting microbial metabolites could help overcome challenges in lung cancer immunotherapy, reduce adverse effects, and involve approaches like fecal microbiota transplantation, microecological therapies, the synthesis of metabolites, and drugs aimed at metabolic pathways.^185^ *Streptococcus pneumonia* is a commensal and pathogenic bacterium in the lungs. Its toxins, pneumolysin and pyruvate oxidase, can dephosphorylate histone H3 at serine position 10 through the host’s PP1 phosphatase, aiding its colonization and growth. This suggests that commensal *Streptococcus* species may help regulate H3S10 phosphorylation levels, which, when elevated, are linked to cancer initiation and progression. While it is clear that the microbiome plays a significant role in shaping host epigenetics, a better understanding of how microbiome dysbiosis disrupts this balance and contributes to lung cancer is needed.^186–188^ The mechanisms of gut microbiota dysbiosis causing lung cancer mediated through various pathways are illustrated in Figure 4.

## Signaling pathways involved in lung cancer

Recent advancements in lung cancer biology have paved the way for personalized treatments targeting genes and pathways. In lung cancer, numerous pathways and their key components undergo functional alterations, and these pathways are increasingly recognized for their critical role in targeted therapies. Notably, key signaling pathways that hold promise as therapeutic targets include those that promote tumor growth, such as the epidermal growth factor receptor (EGFR), Ras, and phosphatidylinositol 3-kinase (PI3K) pathways. Additionally, pathways that inhibit growth, such as the p53/retinoblastoma protein (Rb)/alternative reading frame protein(p14ARF) and serine/threonine kinase 11 (STK11) pathways, as well as apoptotic pathways involving Bcl-2, Bax, Fas, and FasL, are also important. Furthermore, genes responsible for DNA repair and cellular immortality are emerging as key players in developing effective targeted therapies.^189^

Recent experimental studies conducted on animal models have explored how probiotics target PI3K/Akt/mTOR signaling, autophagy, and markers of inflammation. These investigations have uncovered a new mechanism that explains how probiotics can reduce the expression of pro-inflammatory cytokines such as IL-1β, IL-6, and TNF-α. This effect is linked to a decrease in mTOR/FOXO1/NF-κB activity and the enhancement of autophagy processes involved in normal lipid biosynthesis.^190^ Both *in-vitro* and *in-vivo* research have supported these findings, revealing that certain probiotic strains (like *Bacillus amyloliquefaciens* SC06 and *Bacillus licheniformis* SC08) can mitigate oxidative stress by activating the intestinal autophagy system through the inhibition of the PI3K/Akt pathway.^191^ Numerous studies have demonstrated that both intrinsic and extrinsic apoptosis pathways play crucial roles in tumor regression, with regulation occurring through signaling pathways such as PI3K/Akt, p38 MAPK, c-Jun N-terminal kinase (JNK), and AMPK.^192,193^ Research indicates that *Lactobacillus rhamnosus* GG and its metabolites can protect human and mouse intestinal epithelial cells from cytokine-induced apoptosis by inhibiting p38 MAPK activation and promoting the PI3K/Akt pathway.^194^ Similarly, both live and heat-inactivated *Bifidobacterium animalis* subsp. *lactis* BI-04 has been shown to reduce Benzo(a)pyrene (BaP)-induced apoptosis in colonic epithelial cells, acting through the upregulation of the PI3K/Akt pathway and downregulation of p53 gene expression.^195^ Some researchers have also proposed that heat-killed *Saccharomyces cerevisiae* can trigger apoptosis in the SW480 cell line by enhancing the expression of pro-apoptotic proteins like BAX and cleaved caspases-3 and − 9, while simultaneously decreasing the levels of p-Akt1, Bcl-XL, and pro-caspases 3 and 9, which are associated with the Akt/NF-κB pathway. Based on these findings, targeting apoptosis and survival pathways through probiotics presents a promising approach for cancer therapy.^196^

## Probiotics and gut microbiota in lung cancer therapy

**Figure 5.**
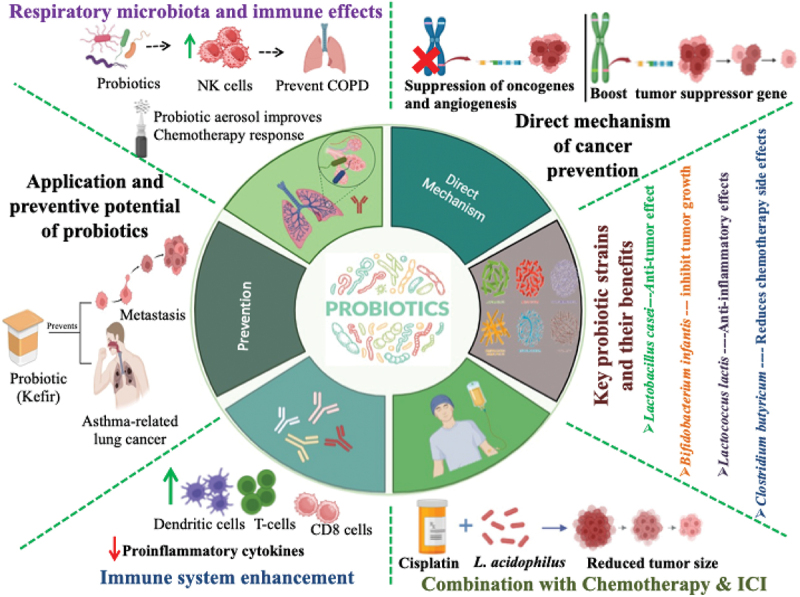
Therapeutic potential of probiotics in lung cancer care through gut microbiota interaction. This figure illustrates the mechanisms through which probiotics may contribute to preventing and treating lung cancer. Probiotics, defined as beneficial live microorganisms, promote immune regulation and enhance gut-lung interactions, potentially offering therapeutic benefits in lung cancer management. Certain probiotic strains, such as *lactobacillus casei* and *bifidobacterium infantis*, have demonstrated antitumor effects and may enhance treatment outcomes when used in conjunction with conventional therapies. Research indicates that probiotics can improve immune responses, mitigate chemotherapy-induced side effects, and reduce heavy metal absorption, which may lower the risk of lung cancer. As research progresses, probiotics are emerging as promising complementary treatments for lung cancer prevention and therapy. Illustration created with Biorender.com.

Abbreviations: NK cells, Natural killer cells; COPD, Chronic obstructive pulmonary disease.

Probiotics are beneficial live microorganisms that enhance the immune system when taken in sufficient amounts. They are believed to support immune regulation by producing various metabolites that affect the gut and distant organs, including the lungs, via the bloodstream. Numerous randomized controlled trials have underscored the essential role of probiotics in promoting gut health, particularly in preventing and treating lung cancer. The concept of managing immune responses and maintaining lung balance through beneficial bacteria is supported by the use of probiotics, which enhance the gut-lung interaction.^1^ Extensive research indicates that probiotics are crucial in preventing and treating various types of tumors.^197^ Research into the anticancer potential of probiotics began several decades ago. Matsuzaki et al. provided the first evidence of probiotics helping combat lung cancer, sparking further investigations with encouraging results. In 1985, they demonstrated the antitumor effects of *Lactobacillus casei* through animal studies using Lewis lung carcinoma cells and line-10 hepatoma. Their experiments on mice and guinea pigs showed that *L. casei* strains effectively reduced lung metastases and cancer spread to nearby lymph nodes.^198^ Further, in 1991, Kim and colleagues explored the anti-cancer effects of seven probiotic strains—*Lactobacillus casei*, *L. plantarum*, *L. acidophilus*, *L. bulgaricus*, *Leuconostoc mesenteroides*, *Streptococcus thermophilus*, and *Bifidobacterium bifidum*—on sarcoma 180 cells and mouse Lewis lung cancer cells. These probiotics were administered via intra-peritoneal injection, and their effectiveness was measured by improved body weight and increased survival time. Among the tested strains, *Lactobacilluscasei* showed the most promising results.^199^ Aragon et al. reported that milk fermented with *Lactobacillus casei* strains exhibited anti-tumor effects in a breast cancer mouse model, suppressing tumor growth, decreasing lung metastasis, and reducing blood vessel formation. Additionally, the probiotic improved immune responses by increasing CD8+ and CD4+ lymphocytes while reducing macrophage infiltration in tumors and lungs.^200^ These findings support the fact that *Lactobacillus casei* have an antitumor effect and may be useful in treating lung cancer. In a study involving metastatic Lewis lung carcinoma (3LL) and solid sarcoma 37 (S37) models, combined therapy effectively reduced metastasis. This approach involves an anti-tumor vaccine, cytotoxic lectin derived from *Bacillus subtilis*, and a probiotic blend containing *Saccharomyces cerevisiae* and *Enterococcus faecium* (or their metabolites). The combination therapy produced a synergistic effect, showing 2 to 2.5 times greater metastasis inhibition than animals treated with the vaccine alone. These findings highlight the enhanced potential of integrating probiotics with conventional cancer treatments.^201^ Zhu et al. developed a system to transfer the sFlt-1 gene using *Bifidobacterium infantis* through electroporation and tested its anti-tumor effects on Lewis lung cancer (LLC) in mice. The soluble fms-like tyrosine kinase receptor (sFlt-1) is a soluble form of the extracellular domain of vascular endothelial growth factor receptor-1 (VEGFR-1) known for its antitumor properties. In another study, a gene transfer system using *Bifidobacterium infantis* successfully expressed sFlt-1 at both the gene and protein levels. This system effectively inhibited the VEGF-induced growth of human umbilical vein endothelial cells *in-vitro*. Additionally, it significantly slowed tumor growth and extended the survival of LLC C57BL/6 mice. These findings highlight the potential of the *B. infantis*-mediated sFlt-1 gene transfer system as a promising therapeutic strategy for treating lung cancer.^202^ Researchers developed a prokaryotic expression system for the soluble kinase insert domain receptor (sKDR) using *Bifidobacterium infantis*. They tested the approach on three groups of mice with Lewis lung cancer (LLC). Group A received saline, Group B was treated with *B. infantis* containing the pTRKH2-PsT plasmid, and Group C received *Bifidobacterium infantis* with the pTRKH2-PsT/sKDR plasmid. The results showed that mice in Group C had better quality of life and longer survival than the other groups. This recombinant *Bifidobacterium infantis* also enhanced tumor suppression by increasing tumor necrosis and extending survival time in the LLC C57BL/6 mice. Additionally, the study demonstrated an anti-angiogenesis effect through an MTT assay performed *in-vitro*, highlighting its potential to limit blood vessel formation in tumors.^203^ These studies highlight the promising potential of *Bifidobacterium infantis* as a new tool in cancer treatment. By acting as a delivery system for anti-tumor genes, it helps block the formation of blood vessels that feed tumors, slowing their growth. The findings also show that mice treated with *Bifidobacterium infantis*-based therapies lived longer and experienced better quality of life. This innovative approach could complement traditional treatments like chemotherapy, offering new hope for more effective cancer care. However, more research is needed to refine this strategy and explore how it might benefit patients with different types of cancer. Mlu et al. found that combining probiotics such as *Lactobacillus*, *Bifidobacterium*, and *Bacteroides* with chemotherapy improved gut health and reduced gastrointestinal complications in lung cancer patients, supporting the complementary use of probiotics in cancer care.^204^ Cheng et al. found that excessive antibiotic use made mice more vulnerable to developing aggressive tumors, such as engrafted B16/F10 melanoma and Lewis lung carcinoma, with reduced survival rates. This was linked to lung microbiota disruptions, which weakened the immune system and impaired natural defense mechanisms. The antibiotic-treated mice exhibited larger, more aggressive tumors, attributed to a failure in triggering an effective γδT17 cell response and the establishment of a tumor-friendly microenvironment in their lungs. Researchers reintroduced normal γδT cells or IL-17 cells to reverse these immune deficiencies, which helped restore immune function. This study highlights the essential role of commensal bacteria in maintaining immune balance and underscores their importance in cancer prevention and treatment.^205^ Han et al. investigated the anti-cancer properties of *Lactococcus lactis* against several cancer cell lines, including human lung, colon, and breast cancer. The strain demonstrated strong anti-inflammatory activity, inhibiting cancer cell proliferation and reducing nitric oxide and pro-inflammatory cytokine production, suggesting potential use for cancer management.^206^ Another study by Lee et al. demonstrated the anti-cancer properties of *Lactococcus lactis* on various cancer cell lines, including lung, colon, and breast cancers, noting significant inhibition of cell proliferation. Their research further supports the role of probiotics in cancer management.^207^ Zamberi et al. explored the potential of Kefir, a fermented milk product containing probiotics, in fighting cancer. Their study, using 4T1 breast cancer cells and BALB/c mice, demonstrated Kefir’s cytotoxic effects. Notably, Kefir enhanced the activity of T helper and cytotoxic T cells while significantly reducing cancer metastasis to the lungs and bone marrow.^208^ An *in-vivo* study using Lewis lung cancer (LLC)-bearing mice (C57BL/6J) explored the impact of probiotics on cancer treatment. The mice were divided into three treatment groups: one receiving cisplatin alone, another receiving cisplatin with an antibiotic cocktail (Vancomycin, Ampicillin, and Neomycin, which disrupts gut microbiota balance), and the third receiving cisplatin alongside *Lactobacillus acidophilus* (a probiotic strain). The results were promising – mice treated with both cisplatin and *L. acidophilus* showed smaller tumors and lived longer compared to those in the other two groups. In contrast, mice receiving either cisplatin alone or cisplatin with antibiotics had shorter survival times. Additionally, western blot analysis revealed that the probiotic treatment suppressed oncogenes (VEGFa and Ras) while boosting tumor suppressor genes (Cdkn1b and Bax). The probiotic group also exhibited enhanced anti-tumor immune responses, with increased expression of interferon (IFN)-γ, Granzyme B (Gzmb), and Perforin-1 (Prf1) mRNA. These findings underscore the potential of combining probiotics with chemotherapy to improve cancer treatment outcomes.^209^ Sivan et al. reported that oral administration of *Bifidobacterium* strains (including *B. longum*, *B. lactis*, and *B. breve*) in cancer-induced mice promoted anti-tumor immunity and enhanced immune responses, such as CD8+ T-cell activation and improved dendritic cell function. Their findings highlight the potential of probiotics in supporting immunotherapy.^210^ Na-Kyoung et al. investigated *Lactococcus lactis* isolated from Kimchi and found it to be effective against various cancers, including gastric, colon, breast, and lung carcinoma. This probiotic demonstrated antioxidant properties, inhibited pathogen adhesion to the intestinal lining, and showed promising anti-cancer effects, suggesting its potential as a functional food for cancer management.^207^ Lastly, Daillère et al. identified two bacterial species, *Enterococcus hirae* and *Barnesiella intestinihominis*, as “oncomicrobiotics” that enhanced immune responses and prolonged survival in advanced lung cancer patients receiving chemotherapy. These findings highlight the therapeutic potential of microbiota in cancer treatment.^211^ A survey conducted at the outpatient department of the National Cancer Institute in Slovakia explored the use of probiotics among cancer patients and their connection to various tumor characteristics. Patients were asked to complete a questionnaire that covered several aspects, including the duration and method of probiotic use in relation to their cancer treatment, expectations, side effects experienced, awareness of potential risks, dietary supplement habits, and overall experiences with probiotics. Out of 499 participants with different types of cancer, 25 were lung cancer patients. Among them, 60% (15 patients) reported no noticeable benefits from probiotic use, while the remaining 40% (10 patients) had a positive experience. These findings provide insights into the mixed responses to probiotics among cancer patients, highlighting the need for further research to better understand individual outcome variations.^212^ In a recent study, Yang et al. investigated the effects of *Clostridium butyricum* on lung cancer patients undergoing chemotherapy. Following a preplanned protocol, they monitored changes in gut flora by analyzing fecal samples at various stages of treatment. For comparison, placebo trials were also conducted. The findings revealed that patients receiving *C. butyricum* experienced fewer instances of chemotherapy-induced diarrhea, showed reduced inflammation, and maintained better physiological balance.^213^ Yang et al. conducted a pooled data analysis from over 1.44 million individuals across the United States, Europe, and Asia. In this large pooled analysis, it was revealed that both high dietary fiber and yogurt intake were linked to a lower risk of lung cancer, regardless of other known risk factors. Interestingly, the study also found that consuming fiber and yogurt together might offer an even greater protective effect against lung cancer. Their research indicates that prebiotics and probiotics may play a protective role in preventing the development of lung cancer.^19^ In a study published in 2024, researchers explored how adding oral probiotic supplements to immune checkpoint inhibitors (ICIs) could affect treatment outcomes for patients with advanced lung cancer. The study involved 253 participants, some receiving probiotics alongside ICI therapy. The results were promising: patients with small cell lung cancer (SCLC) who took probiotics saw a significant improvement in their progression-free survival (PFS) compared to those who didn’t. There was also a positive trend in PFS among non-small cell lung cancer (NSCLC) patients who received probiotics. Overall, this study suggests that combining oral probiotics with ICIs could improve treatment outcomes for those with advanced SCLC. However, more research is needed to confirm these hopeful findings.^214^ A study highlights the critical role of the gut-lung axis in modulating immune responses and its implications for lung cancer and immunotherapy efficacy. The gut microbiome significantly influences the effectiveness of immune checkpoint inhibitors (ICIs) in lung cancer. Dysbiosis and increased gut permeability contribute to systemic inflammation, impacting tumor progression and treatment response. Furthermore, it elaborates on the gut-lung axis, emphasizing how microbial signatures shape immune homeostasis and therapeutic outcomes. This interconnected pathway highlights the need for a multidisciplinary approach to optimize cancer immunotherapy by targeting gut microbial composition and function.^215^ The gut microbiota plays a key role in shaping the pharmacokinetics and effectiveness of anti-cancer drugs, meaning probiotics can also impact treatment outcomes. However, further research is needed to fully understand how probiotics influence bacterial enzyme activity during chemotherapy.^216^ In addition to their direct effects on lung cancer treatment, probiotics can also contribute indirectly through several mechanisms. These include the prevention of respiratory diseases, the modulation of the host’s immune system, and the enhancement of lung immunity.^197^ For instance, studies have shown that certain probiotic bacteria, such as *Lactobacillus* and *Bifidobacterium*, can help decrease the number of pathogens in the respiratory system. This protective effect is believed to be associated with enhanced activity of immune cells, including natural killer (NK) cells and macrophages in the alveolar region.^217^ Probiotics could also benefit patients with chronic obstructive pulmonary disease (COPD), especially those who experience frequent viral infections. They enhance the activity of natural killer (NK) cells and boost the levels of key mediators that are crucial in managing the inflammatory responses often seen during COPD flare-ups.^218^ This suggests that if chronic obstructive pulmonary disease (COPD) and lung cancer are closely connected at the molecular level, then using probiotics as anti-inflammatory treatments for COPD might also aid in preventing and treating lung cancer. Additionally, recent research has highlighted the significant role of interleukin-17 (IL-17) in lung cancer development, noting that smoking is linked to variations in the IL-17 gene. Studies found that lung cancer occurrence was considerably lower in IL-17 knockout mice with a specific lung K-ras mutation compared to those with a local pulmonary K-ras mutation.^219^ Supporting this, a recent study found that a novel probiotic mixture decreased tumor growth by down-regulating IL-17 and its primary-producing cells.^220^ These probiotics promote the development of T regulatory (Treg) cells and shift the Th1/Th2 balance toward a Th1-dominant state.^221^ Supporting this, one study investigated BALB/c mice sensitive to ovalbumin (OVA) by orally administering six different probiotic strains: *Bifidobacterium breve*, *Bifidobacterium infantis*, *B. animalis*, *Lactobacillus plantarum*, and *Lactobacillus rhamnosus*. After the mice inhaled OVA, their reactions to methacholine were assessed, and pulmonary inflammation was evaluated through bronchoalveolar lavage fluid (BALF) analysis for inflammatory cells and mediators. Among the six strains, those treated with *B. breve* and *L. plantarum* exhibited positive effects, including reduced eosinophil counts in BALF, diminished responses to methacholine, and lower levels of both OVA-specific IgE and IgG1. Additionally, *B. breve* decreased interleukins IL-4, 5, and 10, while *L. plantarum* reduced allergic skin reactions to OVA. The other probiotic strains did not appear to influence these parameters.^222^ These findings indicate that probiotics could be crucial in reducing the risk of lung cancer associated with asthma.^197^ Research has shown that feeding mice the probiotic strain *L. pentosus* enhances natural killer (NK) cell activity, leading to increased production of IFN-λ. This rise in IFN-λ is linked to the action of IL-12 produced by CD11c+ dendritic cells, which interact with the bacteria through Toll-like receptors (TLR) 2 and 4.^223^ A recent review elaborated on how probiotics may exert their anticancer effects by modulating inflammatory responses and enhancing levels of helper T cells and interferon γ.^224^ Probiotics could be crucial in lung cancer prevention by boosting immune functions. For instance, studies indicate that different species of *Bifidobacterium* have greater anti-mutagenic properties against benzopyrene when delivered as cell suspensions rather than as supernatants.^225^ Notably, strains like *B. longum*, *L. rhamnosus*, and *L. plantarum* have demonstrated the capacity to bind heavy metals in laboratory settings.^226^ Based on this evidence, it is plausible that a well-chosen combination of probiotic strains could lower the risk of lung cancer by reducing the absorption of heavy metals and promoting their elimination from the body. Regularly consuming these probiotics may also serve as a preventive strategy against heavy metal poisoning, a known contributor to lung cancer. Supporting this notion, a study highlighted the effectiveness of several probiotic strains in cleaning food and water of heavy metals, toxins, and pathogens.^227^ The findings clearly indicate that probiotics play a vital role as a complementary therapy in preventing and treating lung cancer.^197^ Using biotherapeutics to treat respiratory diseases, including lung cancer, is an innovative healthcare approach. These beneficial microorganisms primarily influence gut microbiota through immune modulation. As we understand how these microbes interact with different organ systems and the immune system expands, designing effective probiotic-based therapies will become more feasible. While clinical trials have yielded promising results, further research is essential to determine the best treatment strategies.^1^ The therapeutic potential of probiotics in lung cancer care through gut microbiota interaction is explained in Figure 5.

## Postbiotics, psychobiotics, and para-probiotics in lung cancer treatment

Postbiotics refer to the byproducts of microorganisms that are no longer alive but still offer health benefits to the body. Foods like kefir, kombucha, yogurt, miso, tempeh, and kimchi are rich in postbiotics. Unlike probiotics, postbiotics provide similar health benefits without introducing live bacteria into the gut.^228–230^ Postbiotics can include a variety of substances, such as fragments of bacterial cell walls, metabolites, bacterial lysates, extracellular vesicles, and short-chain fatty acids.^231^ Postbiotics and their bioactive compounds offer many benefits, including improving gut health, regulating the immune system, and helping fight cancer. They can also enhance the effectiveness of cancer treatments while reducing the side effects of conventional therapies, such as vomiting and diarrhea.^232^ In 2024, a study demonstrating the beneficial effects of heat-killed *Lacticaseibacillus paracasei* MCC1849 on human immune cells was published. This randomized, double-blind, placebo-controlled trial involved 100 healthy adults who were randomly assigned to either the MCC1849 group or a placebo group. Participants consumed a powder containing MCC1849 cells or a placebo powder for four weeks. The results showed that taking MCC1849 stimulated peripheral dendritic cells (DCs) and helped maintain the expression of immune markers like IFN-α, β, and γ during infection-like conditions.^233^ In their study, Subramaniam et al. evaluated the *in vitro* cytotoxic effects of various *Mycobacterium indicus pranii* (MIP) fractions on the cervical cancer cell line CaSki and the lung cancer cell line A549. Only the heat-killed MIP bacteria fraction, which can be considered a postbiotic, demonstrated cytotoxic effects among the different fractions. In this fraction, cell viability was reduced to 24% in CaSki cells and 26% in A549 cells, while the remaining fractions showed no cytotoxic effects. Their study demonstrates that the postbiotic form of MIP is effective against both lung cancer cell line A549 and cervical cancer cell line CaSki and warrants further investigation for potential use in the management of lung cancer.^234^ A randomized, controlled trial was conducted in Chongqing, China, to evaluate the effects of JK5G postbiotics on gut microbiota and immune-related adverse events (irAEs) in non-small-cell lung cancer (NSCLC) patients treated with immune checkpoint inhibitors (ICIs). Stage IIIb-IV NSCLC patients without epidermal growth factor receptor (EGFR), ROS1, and anaplastic lymphoma kinase (ALK) mutations were divided into two groups: the control group received chemotherapy and a placebo. In contrast, the JK5G group received chemotherapy with JK5G postbiotics. At the end of the study, the JK5G group exhibited a better quality of life, enhanced nutrition, fewer symptoms of depression, and a reduced incidence of anemia, decreased lymphocyte counts, loss of appetite, nausea, and asthenia. The gut microbiota of the JK5G group was more balanced, with increased levels of *Faecalibacterium* and *Ruminococcaceae* and higher fecal butyrate. Additionally, lower TNF-α, IL-2, and C-reactive protein (CRP)levels were observed, along with higher CD3+CD4+ T cells and an improved CD4/CD8 ratio. Thus, JK5G postbiotics may reduce irAEs, enhance the quality of life and nutrition, and improve gut microbiota, potentially benefiting advanced NSCLC patients undergoing ICIs.^235^

Experiencing psychological distress, such as anxiety and depression, after lung cancer surgery is quite common, even in the absence of complications. Studies show that over 40% of lung cancer patients go through some form of psychological distress following their procedure.^236^ In a cohort study by Park et al. examining the prevalence of anxiety and depression after lung cancer surgery, it was found that 8% of patients experienced anxiety and 12% had depression before the procedure. After surgery, 9% of patients showed signs of anxiety and 19% experienced depression. The study revealed no significant change in the prevalence of anxiety, but the rate of postoperative depression was notably higher compared to the preoperative levels. Thus, careful psychological evaluation and appropriate management are required to improve patient’s quality of life after lung cancer surgery.^237^ In the management of postoperative depression in lung cancer patients, psychobiotics may play a vital role. Psychobiotics are probiotics that can improve mental health when taken in specific amounts, interacting with the gut’s natural bacteria. While the exact ways these bacteria provide benefits aren’t fully clear, it’s thought that they mainly work through the body’s stress response system, hypothalamic-pituitary-adrenal axis (HPA axis), by influencing the immune system and inflammation, and by producing chemicals that affect brain function, like neurohormones and neurotransmitters.^238^ Clinical trial data regarding psychobiotics reducing depression includes *Bifidobacterium longum* NCC3001, at a dose of 1 × 10^10^ CFU/g, which was found to reduce depression scores on the Hospital Anxiety and Depression Scale and reduced responses to negative emotional stimuli in multiple brain areas. The possible mechanism of action is the release of neuroactive compounds through vagal signaling and brain-derived neurotrophic factor (BDNF) regulation.^239^ *Clostridium butyricum* MIYAIRI 588, at a dose of 60 mg/day in combination with antidepressants, is effective in the treatment of treatment-resistant major depressive disorder through the regulation of proinflammatory agents.^240^

Paraprobiotics, also known as parabiotics, dead probiotics, or inactivated probiotics, refer to non-living bacteria that can improve human health. These include components like bacterial cell structures, cell lysates, and bacterial fractions. Typically, paraprobiotics consist of substances such as peptidoglycan, teichoic acid, cell wall polysaccharides, and proteins found on the cell surface. They are known for various beneficial effects, including anti-inflammatory, antioxidant, anti-cancer, immunomodulatory, cholesterol-lowering, anti-obesity, and blood pressure-reducing properties.^241–243^ Some commonly used para-probiotic strains include but are not limited to *Lacticaseibacillus casei*^244^. *Lactobacillus rhamnosus GG*.^245^ *Saccharomyces boulardii*.^246^ Brandão et al. demonstrated that inactivated *Lacticaseibacilluscasei* bacteria can influence the intestinal microbiota of mice by boosting the abundance of beneficial bacteria like *Lachnospiraceae*and *Ruminoccocaceae*, while decreasing the presence of harmful species such as *Clostridiaceae, Enterobacteriaceae*, and *Helicobacteriaceae*.^244^ Arai et al. found in their study that when *L. paracasei* paraprobiotic is orally given to mice, it stimulates the production of antigen-specific IgA in the small intestine, serum, and lungs while also boosting the percentage of follicular helper T cells in the Peyer’s patches.^247^ Administering *Enterococcus faecalis* paraprobiotic for one week before chemotherapy helped minimize intestinal damage in the ileum of mice treated with irinotecan. It also lowered the levels of neutrophils and macrophages and prevented bacteremia. This effect was linked to preserving *zonula occludens* protein integrity.^248^ The research conducted by Hwang et al. highlights the anticancer effects of heat-killed *Lactobacillus brevis* KU15176 (KU15176). The findings showed that KU15176 led to increased expression of apoptosis-related genes (Bax, caspase-3, and caspase-9), DNA damage, a higher apoptosis rate, and enhanced caspase activity in the human gastric cancer cell line (AGS). These results point to the potential of KU15176 as a preventative agent against cancer.^249^ These studies support the use of paraprobiotics as a potential strategy for managing cancer and side effects caused by chemotherapy and radiation therapy. They could offer similar benefits to probiotics while potentially having a safer profile. The potential role of postbiotics, psychobiotics, and paraprobiotics in managing lung cancer has been outlined above. However, further research is crucial to fully understand their effectiveness and facilitate their widespread use in lung cancer treatment.

## Role of polyphenols in combatting lung cancer through gut microbiota interaction

Polyphenols are a diverse group of over 40,000 natural compounds found in plants. They help plants defend themselves against pathogens and environmental stress while also playing a role in interactions with other organisms. When we consume plant-based foods like fruits and vegetables, the polyphenols they contain may lower the risk of various cancers.^250^ Recent clinical studies have shown that polyphenols can positively influence gut microbiota in healthy individuals by promoting the growth of beneficial microbes. Specifically, intervention trials reported increased fecal levels of *Bifidobacterium* and *Lactobacillus*, both following the consumption of polyphenol-rich foods or extracts. These included de-alcoholized red wine,^251^ green tea, and blueberry drinks, demonstrating the microbiota-enhancing effects of these polyphenol sources.^252,253^ A diet rich in fruits and vegetables may aid in lung cancer prevention due to its high levels of antioxidant polyphenols, including flavonoids, proanthocyanidins, lignans, stilbenes, and phenolic acids. In addition to their antioxidant role, these polyphenols help regulate cellular pathways, promote cell survival, and provide strong anticarcinogenic and antimutagenic benefits. Polyphenols exhibit three key functions: acting as powerful antioxidants, modulating phase I and II enzymes, and regulating cell survival pathways to protect against lung cancer development.^42^ Soria et al. outlined three key chemopreventive strategies for polyphenols: Primary prevention, which focuses on reducing cancer risk in healthy individuals with a high susceptibility; Secondary prevention, aimed at stopping the progression of precancerous lesions into cancer; and Tertiary prevention, which helps prevent recurrence or metastasis in individuals with a history of cancer.^254^ In their study, Yuan et al. utilized PICRUSt analysisof16S rRNA sequencing in fecal samples and found that green tea consumption reduced microbial genes involved in inflammation-related pathways, such as those responsible for lipopolysaccharide (LPS) biosynthesis, a key driver of inflammation.^253^ Research in both humans and animals suggests that polyphenols can selectively promote the growth of specific gut microbes. However, the precise mechanisms driving these effects remain unclear. Current evidence points to a mutual interaction between polyphenols and gut microbes. On one hand, polyphenols can influence bacterial growth by inhibiting nucleotide synthesis, binding to essential metal ions, interacting with cell membranes, or supporting electron transfer processes crucial for bacterial energy production. On the other hand, gut microbes play a role in breaking down polyphenols, which, in turn, can reshape the microbial community, further enhancing their health benefits.^255^ Since gut microbes significantly metabolize polyphenols, several studies have explored the anticancer potential of these microbial metabolites. For example, Wang et al. identified Baicalein as a key metabolite derived from baicalin, a compound found in *Scutellaria baicalensis* extract. They discovered that baicalein exhibited stronger antiproliferative and pro-apoptotic effects than baicalin, along with a greater ability to arrest the cell cycle at the S phase in colon cancer cells. In a nude mouse model, baicalein also demonstrated superior tumor inhibition than baicalin.^256^ Epigallocatechin-3-gallate (EGCG), a prominent polyphenol found in green tea, has been shown to protect cells from the harmful effects of hexavalent chromium [Cr(VI)]. In a dose-dependent manner, EGCG reduces Cr(VI)-induced apoptosis by inhibiting the activation of key cell death mediators, such as caspase-3 and nuclear poly (ADP-ribose) polymerase (PARP). Additionally, it decreases the production of intracellular reactive oxygen species (ROS) and minimizes DNA-protein cross-links, helping to preserve cellular integrity.^257^ It has been proven that bisdemethoxycurcumin, a compound derived from Curcuma longa (turmeric), prevents premature aging in lung fibroblast cells (WI-38) by activating the Sirt1/AMPK signaling pathway when exposed to oxidative stress from tert-butyl hydroperoxide (t-BHP).^258^ Luteolin, a polyphenol found in fruits like kiwi and melons, was shown to reduce cigarette smoke extract (CSE)-induced damage in normal human bronchial epithelial cells (NHBE). It decreased cell death and suppressed the expression of Nrf2, NQO1, and HO-1 enzymes, all associated with oxidative stress. Moreover, luteolin increased glutathione levels, which further curbed the generation of harmful reactive oxygen species (ROS).^259^ Flavonols such as Quercetin and Kaempferol, abundant in apples and onions, also exhibit protective effects. Quercetin has been shown to inhibit phase I enzymes (CYP1A1 and CYP1B1) induced by benzo[a]pyrene (BaP), a carcinogen found in tobacco smoke. Kaempferol reduces the activation of phase I enzymes triggered by cigarette smoke condensate (CSC) and prevents CSC-induced cellular transformations, lowering the risk of cancer development.^260^ Quercetin also modulates cellular redox balance by activating the Nrf2-driven heme oxygenase-1 (HO-1) pathway, further highlighting its antioxidative properties.^261^ Similarly, caffeic acid, another polyphenol, scavenges ROS and prevents lipid peroxidation in fibroblast cells exposed to hydrogen peroxide (H₂O₂). It boosts antioxidant enzyme activity, like catalase, to protect cells from oxidative damage.^262^ Resveratrol, commonly found in grapes, and methoxylated flavonoids have been shown to inhibit the activation of CYP1A1 proteins and block DNA binding by harmful carcinogens like benzo(a)pyrene (BaP).^263^ In experimental studies using animal models, Mangiferin, a polyphenol found in mangoes, demonstrated protective effects against BaP-induced lung cancer by enhancing phase II detoxification enzymes.^264^ In Shanghai, a study found that nonsmoking women who regularly consumed green tea had a 35% lower risk of developing lung cancer compared to non-tea drinkers.^265^ Other studies found that certain bioactive compounds, such as β-cryptoxanthin from citrus fruits, were associated with a reduced risk of lung cancer, even among smokers. Similarly, a study in Hawaii demonstrated an inverse relationship between lung cancer risk and the regular consumption of flavonoid-rich foods like onions, apples, and grapefruit. Notably, onions were particularly effective in reducing the risk of squamous cell carcinoma by inhibiting cytochrome P450 enzymes that activate pro-carcinogens.^42^ A large European cohort study involving 521,468 participants across 10 countries found that smokers who consumed various fruits and vegetables had a reduced risk of squamous cell carcinoma. However, no significant link was found for adenocarcinoma or small cell carcinoma. This suggests that consuming a diverse range of fruits and vegetables provides a variety of bioactive compounds that work synergistically to lower cancer risk.^266^ The research underscores the importance of a healthy, plant-based diet rich in polyphenols and diverse fruits and vegetables for reducing lung cancer risk, especially among smokers. These bioactive compounds inhibit carcinogen activation and enhance the body’s antioxidant defenses, highlighting the potential of dietary interventions in cancer prevention. However, not all microbial metabolites enhance anticancer effects. For instance, Abd-Rabou et al. found that, following *in-vitro* fermentation, the antiproliferative activity of a palm date polyphenol extract in Caco-2 cells dropped by more than twofold.^130^ This reduction could be attributed to either a decline in the concentration of active compounds or the formation of inactive metabolites. These studies emphasize the importance of understanding how microbial metabolism influences polyphenol activity. Future research should focus on analyzing polyphenol metabolites alongside changes in gut microbiota and disease outcomes to better comprehend the interplay between polyphenols, microbes, and their anticancer effects.^255^

## Dietary fibers and gut microbiota in lung cancer management

Studies revealed that both the quantity and quality of carbohydrates have a significant impact on lung cancer risk, particularly high dietary glycemic index (GI) compared to low dietary glycemic load (GL) being positively correlated with the disease. Low-GI carbs found in natural products like fruits, vegetables, and whole grains were generally considered to be healthier than high-GI carbohydrates found in highly processed cookies, cakes, and beverages with sugar. In a dose-dependent fashion, eating more whole grains was substantially linked to a decreased risk of lung tumors across men and women. More precisely, eating 2.3 servings of whole grains daily compared to 0.3 servings was associated with a decreased risk of lung cancer.^267^ One possible biological explanation is that foods with a high GI can cause an immediate spike in blood sugar, which in turn initiates the release of insulin and insulin-like growth factors (IGFs). These factors regulate DNA synthesis via the tumorigenesis-related mammalian target of rapamycin (mTOR) pathways and phosphatidylinositol-3 kinase (PI3K).^268^ Short-chain fatty acids (SCFAs) are created when gut microorganisms ferment dietary fiber as they navigate the large intestines during digestion. As part of our body’s anticancer protective system, these SCFAs can boost immunological responses, alter anti-inflammatory pathways, regulate insulin secretion, and promote the production of cytokines.^120^ Likewise, SCFAs are vital sources of nourishment for establishing gut microbiota, which can affect immunological response and respiratory inflammation via the gut-lung axis.^120,267^ As per a recent meta-analysis and systematic review, whole grains high in fiber in the diet were analogously linked to a 27% reduced likelihood of lung cancer.^269^ It is crucial to highlight the role of fiber and carbohydrates from natural sources such as fruits, vegetables, and whole grains in preventing lung cancer. Studies suggest that consuming dietary fiber exceeding 25 g/day is protective against chronic diseases. Specifically, individuals with an average intake of 30.5 g/day – the highest quartile of fiber consumption – showed the lowest risk of developing lung cancer. Conversely, a higher intake of carbohydrates from sugary beverages is associated with an increased likelihood of lung cancer.^267^

**Figure 6.**
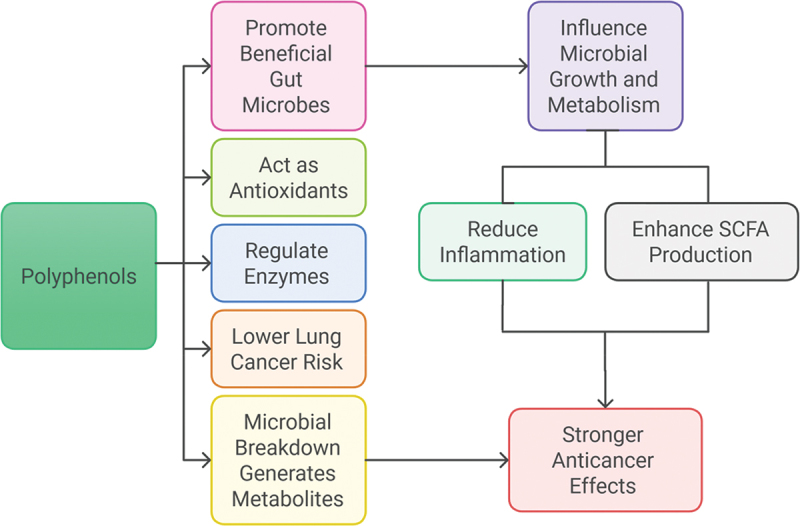
Role of polyphenols in combatting lung cancer through gut microbiota interaction. This figure illustrates the role of polyphenols in cancer prevention and treatment, with a focus on lung cancer. Polyphenols have been shown to reduce the risk of lung cancer and improve gut health by promoting beneficial gut microbiota, such as *bifidobacterium* and *Lactobacillus*. These polyphenols help strengthen the gut microbiota, influencing microbial growth and metabolism, reducing inflammation and enhancing short-chain fatty acid (SCFA) production. As antioxidants, polyphenols regulate enzymes and protect cells from oxidative damage. Specific polyphenols, such as flavonoids and proanthocyanidins, have demonstrated potential in lowering lung cancer risk. Additionally, microbial breakdown of polyphenols can yield metabolites like baicalein (from baicalin), which exhibit stronger anticancer effects than their parent compounds. However, some studies suggest that the microbial metabolism of polyphenols may reduce their anticancer efficacy, highlighting the importance of studying polyphenol metabolites for a deeper understanding of their biological effects. This figure underscores the significance of polyphenol-rich diets in cancer prevention and emphasizes the crucial role of gut microbiota in modifying or enhancing these effects.

Dietary fiber is indigestible to humans, but the gut microbiota can ferment it to produce SCFAs.^270^ Recent research indicates that SCFAs’ positive effects on metabolism and the host immune system extend beyond the gut to other organs, including the airways.^49,270^ Further, probiotics function as an immunomodulator by regulating the release of cytokines and the growth and development of immune cells. Yogurt is believed to aid in preventing lung disorders, as both *in-vitro* and *in-vivo* studies have shown that specific probiotic strains possess anticancer and anti-inflammatory properties, suppress lung metastasis, and enhance natural killer cell activity.^197,271^ According to a current study, there was a stronger negative correlation between the risk of lung cancer and yogurt intake and dietary fiber in participants with squamous cell carcinoma and those with proinflammatory conditions (such as heavy alcohol users), indicating that these foods may have a positive impact on lung carcinogenesis through anti-inflammatory mechanisms.^19^ Emerging evidence has also suggested a synergistic effect of probiotics and prebiotics on host health. Fermentation of prebiotics can encourage the colonization of probiotic bacteria that promote health, like *Lactobacillus* and *Bifidobacterium*, in the gastrointestinal tract,^272^ which can enhance the gut microbial ecosystem and increase the physiological benefits of bacteria. The results of our current study suggested that a combination of probiotics (yogurt) and prebiotics (fiber) may be more effective against cancer of the lungs than either one alone.^19^ By decreasing lipid oxidation, raising levels of the anti-inflammatory cytokine adiponectin and lowering blood glucose concentrations, dietary fiber eradicates inflammation. Further evidence of dietary fiber’s anti-inflammatory qualities comes from studies showing that a high fiber intake lowers the level of the systemic inflammation marker C-reactive protein.^273,274^ The role of polyphenols in combatting lung cancer through gut microbiota interaction is explained in Figure 6.

## Microbiome-targeted interventions in cancer: personalized approaches

Individual differences in humans, influenced by genetic and environmental factors, make creating effective population-wide approaches for early disease detection, treatment, and prognosis challenging.^275^ Personalized medicine customizes treatments based on the unique characteristics of each patient, presenting a promising approach to managing microbiome-related health concerns. Notable approaches include fecal microbiota transplantation (FMT), where microbes from healthy donors are introduced into patients, showing potential for treating conditions like recurrent *Clostridium difficile* infection.^276^ Multiple studies have demonstrated that fecal microbiota transplantation (FMT) effectively treats conditions like inflammatory bowel disease, irritable bowel syndrome, and hepatic encephalopathy.^277–279^ More recently, FMT has shown positive results in patients who do not respond to immune checkpoint inhibitor (ICI) therapy, positioning FMT as a promising new strategy in cancer treatment.^280^ In a study by Routy et al., fecal microbiota transplantation from responder patients to non-responder cancer patients receiving ICIs was conducted, and it was observed that the non-responder patients converted to responders with increased diversity of gut microbiota and enhanced antitumor immune response.^281^ Furthermore, probiotics, which are live microorganisms similar to beneficial gut bacteria, and prebiotics, non-digestible substances that support microbial growth, are gaining traction as methods to restore microbiome balance. When combined, these strategies form synbiotics, which enhance microbial colonization and therapeutic benefits.^276^ A growing number of case studies are adding to the expanding evidence supporting the effectiveness of personalized probiotics in cancer immunotherapy. Gopalakrishnan and colleagues investigated the effects of orally administered personalized probiotics, tailored to an individual’s gut microbiota composition, in combination with immune checkpoint inhibitors (ICIs) for cancer patients. The results demonstrated an improved response to ICIs, enhanced antitumor immunity, and increased infiltration of cytotoxic T lymphocytes into tumors.^282^ Advances in metagenomics and sequencing technologies allow for a deeper understanding of individual microbiome profiles, paving the way for personalized interventions.^276^

## Clinical trials on the treatment of lung cancer by gut microbiota modulation

Clinical studies on the role of various nutraceuticals like probiotics, polyphenols and dietary fibers in lung cancer treatment were searched from the *clinical trials.gov* repository for clinical studies. A cohort study evaluated prophylactic strategies to manage dermatologic and gastrointestinal side effects associated with Dacomitinib use in advanced non-small cell lung cancer, with probiotics being among the interventions tested.^283^ A study is currently underway in Turkiye to evaluate the impact of a probiotic supplement containing *Bifidobacterium animalis* spp. *lactis* on clinical objective responses, clinical benefit rates, and intestinal microbiota in patients with metastatic non-small cell lung cancer (mNSCLC) undergoing Nivolumab treatment. The findings could pave the way for developing targeted probiotic supplements as an adjunctive therapy for mNSCLC management.^284^ A randomized controlled trial assessed the impact of delayed versus simultaneous initiation of vitamin B_12_ and folic acid supplementation on Pemetrexed-induced hematological toxicity. The study compared patients who began chemotherapy 5–7 days after starting supplementation to those who initiated supplementation within 24 hours of starting chemotherapy.^285^ The clinical implications of probiotics, polyphenols, and various dietary supplements in patients with lung cancer have been emphasized in numerous studies and summarized in a tabular format in Table 2.^285–293^

**Table 2.** Clinical trial studies examining the effects of various dietary supplements on lung cancer treatment outcomes.

Sl. No	Trial identifier	Title	Details	Status	Reference
1.	NCT01465802	Archer 1042: a phase II study of dacomitinib in advanced NSCLC (post-chemotherapy or select first line patients) to evaluate prophylactic intervention on dermatologic and gastrointestinal adverse events and patient reported outcomes	This study evaluates the impact of prophylactic treatment and an interrupted dosing schedule on adverse events in advanced NSCLC patients receiving dacomitinib, particularly those with EGFR or HER mutations.	Completed	^283^
2.	NCT06428422	The impact of probiotic on survival and treatment response in metastatic NSCLC patients.	This study explores the impact of *Bifidobacterium animalis lactis* BL-04 on clinical outcomes and gut microbiota in Turkish metastatic NSCLC patients receiving nivolumab, aiming to develop probiotics as complementary cancer immunotherapy.	Recruiting	^284^
3.	NCT02679443	Optimum duration of vitamin B12 and folate supplementation in non-squamous NSCLC patients undergoing pemetrexed containing chemotherapy: a randomized controlled trial	Patients will receive a chemotherapy regimen of pemetrexed and cisplatin (or carboplatin for intolerant patients) with dose modifications based on toxicity. Supplementation includes folic acid, iron, and vitamin B12, given daily during chemotherapy and for at least 3 weeks post-treatment.	Completed	^286^
4.	NCT05094167	The mechanism of probiotic *Lactobacillus Bifidobacterium* V9 (Kex02) improving the efficacy of carilizumab combined with platinum in NSCLC patients	Main focus of the study in this trial is to investigate the role of the gut microbiome in modulating the efficacy of anti-programmed cell death-1 immunotherapy for cancer patients.	Unknown	^287^
5.	NCT04699721	Clinical study of neoadjuvant chemotherapy and immunotherapy combined with probiotics in patients with resectable NSCLC	This study evaluates the safety and effect of neoadjuvant chemotherapy and immunotherapy combined with probiotics for early resectable NSCLC patients.	Active not Recruiting	^288^
6.	NCT03642548	A prospective multicenter double-blind randomized clinical trial of probiotics combined with chemotherapy in the treatment of patients with advanced NSCLC	This randomized, double-blind trial compares progression-free survival, objective response rate, overall survival, and toxicity in stage IIIB/IV NSCLC patients receiving Bifico or placebo with platinum-based chemotherapy, while analyzing blood, tissue, and fecal biomarkers. Samples are also banked for future research.	Unknown	^285^
7.	NCT04871412	Pioneering pre- and post-operative integrative care to improve thoracic cancer quality of care – the thoracic POISE trial – stage III	The thoracic POISE project aims to improve care for thoracic cancer patients by enhancing health-related quality of life, reducing surgical adverse events, prolonging survival, and integrating complementary medicine into surgical oncology care.	Recruiting	^289^
8.	NCT01426620	Salvage therapy with docetaxel and blueberry powder in NSCLC	This study evaluates the feasibility of using blueberry powder rich in anthocyanidins as an adjunct therapy with paclitaxel/docetaxel for NSCLC treatment, based on promising synergistic effects in preclinical data.	Terminated	^290^
9.	NCT03751592	A single arm, open-label, multicenter, phase Ib/IIa studies of chlorogenic acid for injection for safety and efficacy of advanced lung cancer patients	This study aims to evaluate the disease control rate, overall survival, objective response rate, progression-free survival, and ECOG performance status of chlorogenic acid for injection in advanced lung cancer patients.	Unknown	^291^
10.	NCT05805319	Randomized dietary intervention to improve fiber intake in patients with NSCLC treated with ICI therapy	This study evaluates the impact of a nutritionist-guided dietary intervention on increasing fiber intake in NSCLC patients receiving ICI, with personalized recommendations based on the Canada Food Guide. The intervention includes dietary counseling and gradual fiber intake adjustments to achieve a target of 25 g per day, tailored to minimize side effects.	Recruiting	^292^
11.	NCT04175769	Exploring the effects of a nutritional supplement during immunotherapy or combination of immunotherapy and chemotherapy in NSCLC patients	This study investigates the effects of a nutritional product on NSCLC patients’ response to immunotherapy or chemo-immunotherapy, comparing it to a placebo. Participants will take daily capsules, undergo assessments including physical exams, computed tomography scans, and quality of life questionnaires, with data on adverse events and ECOG status.	Recruiting	^293^

Abbreviations: EGFR, Epidermal growth factor receptor; HER, Human epidermal growth factor receptor; NSCLC, Non-small cell lung cancer; POISE, Peri-operative integrative surgical care evaluation; ECOG, Eastern cooperative oncology group; ICI, Immune checkpoint inhibitors.

## Key limitations of microbiome studies in cancer

The growing data on the microbiome’s role in health and disease offers potential for advancing cancer diagnosis and therapy. However, overcoming technological and conceptual challenges is essential to clearly understand host-microbiome interactions. Most microbiome studies use stool or oral samples as accessible representations of the GI microbiome. However, these correlations are limited due to inherent differences between microbiome compositions in stool and those along the GI tract.^294^ In the future, using computational methods to infer the gut microbiome’s community structure from stool samples or through direct, minimally invasive gut sampling could enhance the accuracy and consistency of cancer-microbiome studies. An even greater challenge lies in evaluating the microbiome within tumors themselves. Despite the difficulty in obtaining these samples, microbial signals from tumors could offer valuable clinical insights. For example, the diversity of the tumor microbiome at the time of surgery may serve as a survival predictor for patients with pancreatic cancer.^295^ Although collecting these samples and integrating them into clinical decision-making is a complex task, it holds great potential. It is expected to become a dynamic and valuable research area in the coming years.

Advancements in metabolomics and proteomics have facilitated high-throughput data generation, helping to identify specific microbial compositions and functions linked to cancer progression and the effectiveness of anticancer treatments. A key challenge, however, is the availability of data and resources, which is crucial for improving microbiome research amidst the broader scientific “reproducibility crisis.”.^296^ Another significant challenge is the lack of standardization in data collection and analysis methods, such as DNA extraction techniques or 16S rRNA gene PCRs. This inconsistency makes it difficult to compare, integrate, and analyze datasets from various studies or regions, hindering the generalization of findings. On a positive note, there is a growing trend for microbiome-related research to require the public release of sequencing data and detailed methodologies through platforms like the European Nucleotide Archive or the NIH Sequence Read Archive. However, these datasets often lack comprehensive metadata, primarily due to restrictions from institutional review boards, despite efforts to deidentify patient information. Additionally, information on sample sizes and statistical power calculations is frequently not shared.^297^ The upcoming decade of microbiome research will need improved standardization and data sharing, including the sharing of bioinformatic analysis tools. This will facilitate more consistent processes for denoising, removing host reads, and aligning taxonomic and gene databases.^298^

## Conclusion and perspectives

In conclusion, the gut microbiota and the gut-lung axis play a pivotal role in the development and treatment of lung cancer, opening up exciting possibilities for cancer prevention and therapy through the use of nutraceuticals and microbiota-focused interventions. The relationship between the gut and lungs is bidirectional, with gut microbiota composition and their metabolites significantly influencing immune responses and lung inflammation. This creates new opportunities for treating lung diseases using interventions such as probiotics and fecal microbiota transplantation (FMT). Additionally, gut dysbiosis is closely linked to lung cancer progression and the body’s response to treatment. This suggests that the gut microbiome could serve as both a biomarker and a therapeutic target, potentially improving the effectiveness and reducing the side effects of lung cancer treatments. While nutraceuticals and dietary changes promise to prevent and treat cancer, particularly by influencing gene expression and boosting antioxidant defenses, their benefits can vary greatly depending on individual genetics, lifestyle, and diet. Some supplements may even pose risks, especially for smokers. The use of probiotics in lung cancer treatment is particularly promising, with studies showing they can enhance immune responses, slow tumor growth, improve quality of life, and offer preventive benefits by influencing the gut-lung microbiota connection. However, more research is needed to refine these approaches. A diet rich in polyphenols may also play a role in reducing lung cancer risk by promoting healthy gut microbiota, which helps control inflammation, oxidative stress, and carcinogen activation.

Looking ahead, more research is needed to better understand how gut microbiota and their metabolites interact with the lungs to influence cancer progression and how these processes can be targeted for treatment. Clinical trials are crucial to assess the safety and effectiveness of microbiota-based therapies like probiotics, FMT, and polyphenol-rich diets in lung cancer patients, particularly considering individual genetic and lifestyle factors. As our understanding of the gut-lung axis deepens, it could lead to personalized treatments that integrate nutraceuticals and microbiome-based therapies, offering new opportunities to improve cancer treatment outcomes and enhance patients’ overall quality of life.

## Data Availability

Data sharing not applicable – no new data generated.

## Abbreviations

ACS: American cancer society
ADP: Adenosine diphosphate
ALA: Alpha linolenic acid
AMPK: Adenosine monophosphate activated protein kinase
AP: Activator protein
BA: Bile acids
BALB: Bay area laboratory mouse
BALF: Broncho-alveolar lavage fluid
t-BHP: tert-butyl hydroperoxide
CAM: Complementary and alternative medicine
CCL: Chemokine ligand
CCR: Chemokine receptor
CD: Crohn’s disease
CD8+: Cluster of differentiation 8
CD11+: Cluster of differentiation 11
CDT: Cytolethal distending toxin
CNF: Cytotoxic necrotizing factor
COPD: Chronic obstructive pulmonary disease
COVID: Coronavirus disease
CP: Cyclophosphamide
CRC: Colorectal cancer
CRP: C-reactive protein
CSC: Cigarette smoke condensate
CSE: Cigarette smoke extract
CTLA: Cytotoxic T-lymphocyte-associated protein
CXCL: C-X-C motif chemokine ligand
CYP: Cytochrome P450
DCA: Deoxycholic acid
DDR: Deoxyribonucleic acid damage response
DHA: Docosahexaenoic acid
DNA: Deoxyribonucleic acid
EBV: Epstein – barr virus
EGCG: Epigallocatechin-3-gallate
EPA: Eicosapentaenoic acid
ERK: Extracellular signal-regulated protein kinase
ETBF: Enterotoxigenic *Bacteroides fragilis*
FDA: Food and drug administration
FMT: Fecal microbiota transplantation
GABA: Gamma- aminobutyric acid
GF: Germ-free
GI: Gastrointestinal
GIT: Gastrointestinal tract
GL: Glycemic load
GLA: Gut-lung axis
GLOBOCAN: Global cancer observatory
GM: Gut microbiome
HDAC: Histone deacetylase
HIV: Human immunodeficiency virus
HO-1: Heme oxygenase-1
12,13-diHOME: 12,13-dihydroxy-9Z-octadecenoic acid
HPV: Human papilloma virus
IBD: Inflammatory bowel diseases
ICAM: Intercellular adhesion molecule
ICI: Immune checkpoint inhibitors
IFN: Interferon
IGF: Insulin-like growth factor
IL: Interleukin
ILC: Innate lymphoid cells
JCV: John cunningham virus
JSRV: Jaagsiekte sheep retrovirus
KDR: Kinase insert domain receptor
LC: Lung cancer
LLC: Lewis lung cancer
LPS: Lipopolysaccharides
MAMP: Microbe-associated molecular patterns
MAPK: Mitogen-activated protein kinases
MCPyV: Merkel cell polyomavirus
MTT: 3-(4,5-di-methylthiazol-2-yl)-2,5-di-phenyltetrazolium bromide
MYD88: Myeloid differentiation primary response gene 88
NF-κB: Nuclear factor kappa B
NHBE: Normal human bronchial epithelium
NK: Natural killer
NLR: Neutrophil-to-lymphocyte ratios
NOD: Nucleotide-binding oligomerization domain-like receptors
NQO1: NAD(P)H:quinine oxidoreductase1
NSCLC: Non-small cell lung cancer
OVA: Ovalbumin
PARP: Poly (ADP-ribose) polymerase
PD: Programmed cell death protein
PFS: Progression-free survival
PI: Phosphoinositide
PICRUSt: Phylogenetic investigation of communities by reconstruction of unobserved states
PPARγ: Peroxisome proliferator activated receptor gamma
PRR: Pattern recognition receptor
RNA: Ribonucleic acid
ROS: Reactive oxygen species
SCFA: Short-chain fatty acids
SCLC: Small cell lung cancer
SFB: Segmented filamentous bacteria
STAT: Signal transducer and activator of transcription
TGF: Transforming growth factor
TLR: Toll-like receptor
TNF: Tumor necrosis factor
mTOR: Mammalian target of rapamycin
UC: Ulcerative colitis
VEGF: Vascular endothelial growth factor
